# *ASCL2* induces an immune excluded microenvironment by activating cancer-associated fibroblasts in microsatellite stable colorectal cancer

**DOI:** 10.1038/s41388-023-02806-3

**Published:** 2023-08-17

**Authors:** Dan Zhang, Qi-Qi Ni, Qiao-Yan Liang, Li-Ling He, Bo-Wen Qiu, Ling-Jie Zhang, Ting-Yu Mou, Chen-Chen Le, Yuan Huang, Ting-Ting Li, Shu-Yang Wang, Yan-Qing Ding, Hong-Li Jiao, Ya-Ping Ye

**Affiliations:** 1 Department of Pathology, School of Basic Medical Sciences and Nanfang Hospital, Southern Medical University, Guangzhou, Guangdong China; 2 Guangdong Province Key Laboratory of Molecular Tumor Pathology, Guangzhou, Guangdong China; 3 Department of General Surgery, Nanfang Hospital, Southern Medical University, Guangzhou, Guangdong China

**Keywords:** Colorectal cancer, Cancer immunotherapy, Diagnostic markers

## Abstract

Proficient mismatch repair or microsatellite stable (pMMR/MSS) colorectal cancers (CRCs) are vastly outnumbered by deficient mismatch repair or microsatellite instability-high (dMMR/MSI-H) tumors and lack a response to immune checkpoint inhibitors (ICIs). In this study, we reported two distinct expression patterns of *ASCL2* in pMMR/MSS and dMMR/MSI-H CRCs. *ASCL2* is overexpressed in pMMR/MSS CRCs and maintains a stemness phenotype, accompanied by a lower density of tumor-infiltrating lymphocytes (TILs) than those in dMMR/MSI CRCs. In addition, coadministration of anti-PD-L1 antibodies facilitated T cell infiltration and provoked strong antitumor immunity and tumor regression in the MC38/sh*ASCL2* mouse CRC model. Furthermore, overexpression of ASCL2 was associated with increased *TGFB* levels, which stimulate local Cancer-associated fibroblasts (CAFs) activation, inducing an immune-excluded microenvironment. Consistently, mice with deletion of *Ascl2* specifically in the intestine (*Villin*-*Cre*^+^, *Ascl2* ^*flox/flox*^, named *Ascl2* CKO) revealed fewer activated CAFs and higher proportions of infiltrating CD8^+^ T cells; We further intercrossed *Ascl2* CKO with *Apc*^*Min/+*^ model suggesting that *Ascl2*-deficient expression in intestinal represented an immune infiltrating environment associated with a good prognosis. Together, our findings indicated *ASCL2* induces an immune excluded microenvironment by activating CAFs through transcriptionally activating *TGFB*, and targeting *ASCL2* combined with ICIs could present a therapeutic opportunity for MSS CRCs.

## Introduction

Colorectal cancers (CRCs) show high intratumoral genetic heterogeneity [1, 2], which has a major effect on the efficacy of immunotherapy. A minority (~15%) of CRCs exhibit a microsatellite instability-high (MSI-H) phenotype, a molecular indicator of defective DNA mismatch repair (MMR), and often have sustained responses to immune checkpoint inhibitors (ICIs) due to a higher density of tumor-infiltrating lymphocytes (TILs) [3, 4]. However, the majority (~85%) are microsatellite-stable (MSS) and lack sensitive responses to ICIs [5]. Therefore, elucidating the molecular mechanisms of tumor immune microenvironment remodeling to overcome the unsatisfactory immunotherapy response in patients with pMMR/MSS CRC is urgently needed.

Achaete-scute homolog 2 (*ASCL2*), a basic-helix-loop-helix transcription factor (TF), is a transcriptional target of Wnt signaling [6]. *ASCL2* controls intestinal stem cell fate [7] and is also indispensable for *LGR5*^+^ basal crypt cell dedifferentiation [8]. However, the expression pattern and role of *ASCL2* in intestinal tumors have been controversial. *ASCL2* has been reported to be significantly upregulated in CRCs [9, 10] and promotes the proliferation, metastasis, and chemoresistance of CRC cells [11–13]. Nevertheless, Felipe et al. showed the opposite result: *ASCL2* is silenced by CpG island methylation during CRC progression [14]. Moreover, a transgenic mouse model revealed that ectopic overexpression of *ASCL2* does not increase tumor initiation or progression [15]. A new taxonomy was introduced, which identifies a consensus gene expression–based subtyping classification system for CRC into four consensus molecular subtypes (CMSs). Most MSI tumors are classified as CMS1 and exhibit extensive hypermethylation, dominated by CpG island methylation phenotypes [16]. Our data suggest that these puzzling findings are due to two opposite expression patterns of *ASCL2* in different molecular phenotypes of CRCs. The above contradictions could be explained by analyzing the expression patterns of *ASCL2* in CRCs from different aspects and perspectives.

As a target gene of Wnt signaling, *ASCL2* is strongly upregulated and plays a vital role in maintaining stemness in pMMR/MSS CRCs. A recent study suggested that the stemness index of tumor cells is negatively correlated with the number of infiltrating immune cells in the tumor microenvironment (TME) [17]. Emerging reports have indicated that *ASCL2* plays a crucial role in immune regulation. *ASCL2* has been reported to initiate follicular T helper cell development [18] and promote germinal center B-cell responses by directly regulating AID transcription [19]. *ASCL2* negatively regulated pathogenic Th17 cell differentiation and thus alleviated the intestinal mucosal inflammatory response [20]. In addition, *ASCL2* affected the efficacy of immunotherapy [21] and was associated with immune evasion in pMMR/MSS CRCs [22]. Given the above findings, we speculate that *ASCL2* may be involved in remodeling the immune-excluded microenvironment in pMMR/MSS CRCs.

Cancer-associated fibroblasts (CAFs) are closely associated with highly reactive inflammatory desmoplastic stroma and remodeling of the immune-excluded microenvironment [23]. And evidence of direct interactions between activated CAFs and CD8^+^ T cells was provided, leading to the suppression of T cells [24]. The recruitment of activated CAFs to the tumor parenchyma is governed mainly by the growth factors released by the cancer cells, of which transforming growth factor β (*TGFB*) is a crucial factor [25, 26]. At the same time, *TGFB* promotes the differentiation of Treg cells and inhibits the antitumor ability of CD8^+^ T cells [27], thus attenuating the tumor response to PD-1/PD-L1 blockade [28]. Although targeting the stroma rarely leads to an obvious tumor regression response to chemotherapy and/or immunotherapy [29, 30], the inhibition of *TGFB* is expected to decelerate cancer progression. However, whether *ASCL2* induces CAFs activation to exclude CD8^+^ T cells in pMMR/MSS CRCs has not been reported.

Here, we analyzed the distinct expression patterns of *ASCL2* in pMMR/MSS and dMMR/MSI-H CRCs and explored whether *Ascl2* induces an immune-excluded microenvironment by activating CAFs in pMMR/MSS CRC using intestinal conditional gene knockout mice. Besides, we investigated the feasibility of Wnt pathway inhibitors combined with ICIs treating CRCs with the pMMR/MSS phenotype, which presents a novel strategy and therapeutic opportunity for this disease.

## Materials and methods

### Animal experiments

We crossed *Villin*-*Cre* recombinase transgenic C57BL/6 mice and *Ascl2*^*flox/flox*^ C57BL/6 mice (purchased from Shanghai Model Organisms) and generated intestinal epithelium-specific conditional *Ascl2* knockout mice (*Villin-Cre*^+^, *Ascl2*^*flox/flox*^ mice, abbreviated as *Ascl2* CKO. *Ascl2* CKO and *Apc*^*Min/+*^ mice were generated by breeding *Ascl2* CKO mice with *Apc*^*Min/+*^ mice (purchased from Shanghai Model Organisms). Age- and gender-matched mice were used for further experiments.

For subcutaneous xenograft tumor models, MC38 cells (1 × 10^5^) expressing sh*Ascl2*, *Ascl2*, or scramble control shRNA were subcutaneously injected into the hind limbs of female C57BL/6 mice or Balb/C athymic nude mice. Two weeks (C57BL/6 mice) or four weeks (nude mice) after the operation, the xenografts were excised. Details of the orthotopic implantation mouse model are provided in the Supplementary Materials and Methods. The volume (v) of xenografts was calculated using the following equation: v = π/6 × length × width^2^.

In in vivo studies, female C57BL/6 mice were inoculated subcutaneously in the right flank with 1 × 10^5^ MC38 cells in 100 μL of HBSS (Gibco, 14025092): matrigel (Corning, 356230) (1:1). After 8 days, the mice were randomized into treatment groups and treated the next day with 100 µl of PBS, anti-PD-L1(BioXCell, BE0101) (10 mg/kg first dose followed by 5 mg/kg thereafter), or XAV-939 (Selleck, S1180) (2.5 mg/kg) by intraperitoneal injection. Antibodies were administered three times a week for three weeks. Tumors were measured 2 times per week by a vernier caliper. All mice were randomly selected to receive treatment groups.

C57BL/6, Balb/C athymic nude mice were obtained from the Animal Center of Southern Medical University, Guangzhou, China, and raised under SPF conditions. All mouse experiments were approved by the Laboratory Animal Ethics Committee of Southern Medical University and were instructed in accordance with the good veterinary practice as defined by the Southern Medical University Laboratory Animal Center.

### Clinical tissue specimens

CRC tissues and matched adjacent normal tissues were collected by surgical resection from patients with primary colorectal adenocarcinoma at Nanfang Hospital of Southern Medical University (Guangzhou, China), and none of them received radiotherapy or chemotherapy before surgical removal. Fourteen samples were frozen in liquid nitrogen for qPCR. A total of 82 paraffin-embedded CRC samples were made into a tissue chip for immunofluorescence. A total of 41 paraffin-embedded CRC samples (11 MSI and 30 MSS) were examined for expression of multiple proteins by immunofluorescence. Prior approval was obtained from the Ethics Committee of Nanfang Hospital, Southern Medical University (Guangzhou, China).

### Cell culture

SW480, SW837, RKO, Caco-2, and HCT15, MC38 cells were obtained from the American Type Culture Collection and tested negative for mycoplasma contamination. SW837 and HCT15 cells were cultured in DMEM (Gibco, C11995500BT) with 10% FBS (Vistech, SE100-011), 100 units/mL penicillin, and 100 mg/mL streptomycin; the other cells were grown in RPMI 1640(Gibco, 11875176) with 10% FBS, 100 units/mL penicillin and 100 mg/mL streptomycin. All cells were cultured at 37 °C in a humidified atmosphere of 5% CO2.

### CD8^+^ T cell migration assay

5 × 10^3^ CRC cells were cultured in 50 μL of Matrigel (Corning, 356231) with 50 µl of Organoid Media (Stemcell Technologies, 6005), and with or without 5 × 10^4^ primary human CAFs seeded on the top of Matrigel for 10 days, followed by monitoring 3D tumor spheroid and CAFs growth. Furthermore, activated CD8^+^ T cells (1 × 10^5^ cells/well) were labeled with 1 μM CSFE (MedChemExpress, HY-D0938) for 30 min at 37 °C and then seeded into the upper 5um transwell chamber. After incubation for 12 h, the chamber was removed, and the number of CD8^+^ T cells in the lower chamber was observed directly under a fluorescence microscope.

### Multiplexed immunofluorescence (IF)

Multiplexed IF was performed using the PerkinElmer-Opal-Kit (Akoya Biosciences, NEL811001KT) according to the manufacturer’s instructions. Briefly, the FFPE tissues were dewaxed with xylene, rehydrated through a graded ethanol series, and fixed with 10% neutral-buffered formalin prior to antigen retrieval that was performed with Opal-AR6 Buffer using high-pressure incubation. This step was followed by cooling, blocking, and serial staining with primary antibodies (shown in Supplementary Table 1), HRP-conjugated polymers, and opal fluorophores; cycles were repeated until all markers were stained. Finally, the nuclei were counterstained with DAPI.

### Preparation of single-cell suspension and antibody staining for flow cytometry

Tumors and intestinal epithelial tissue were finely sliced into 0.5–1.0 mm fragments and then enzymatically digested using a cocktail of collagenase IV (Solarbio, C8160), hyaluronidase (Solarbio, H8030) and DNase I (Solarbio, 9003-98-9) for 2 h at 37 °C to obtain a single cell suspension. Single-cell suspensions were filtered through a 40 µm filter. The cells were then stained with the following antibodies for 30 min on ice: CD8-PE (BD Biosciences, 553033), CD4-FITC (Biolegend, 100405), CD25-BV711 (Biolegend, 102049), CD11C-PE (BD Biosciences, 561044), and CD11B-BV605 (Biolegend, 101237). Cells were fixed and permeabilized to stain for FOXP3-PE (TONBO, 50-5773-U025).

Flow cytometry data were collected with a BD Fortessa cell analyzer and analyzed using FlowJo Software (Version 10.8.1, FlowJo).

### Statistical analysis

Statistical analysis was performed with GraphPad Prism V.9 (GraphPad Software) and Fiji. Statistical analyses included Student’s *t* test, Wilcoxon–Mann–Whitney test, and two-way ANOVA. Survival curves were plotted by the Kaplan–Meier method and compared using the log-rank test. *p* < 0.05 was considered statistically significant, *p < 0.05, **p < 0.01, ***p < 0.001, ****p < 0.0001.

## Results

### There are two distinct expression patterns of *ASCL2* in MSS and MSI-H CRCs

**Fig. 1:**
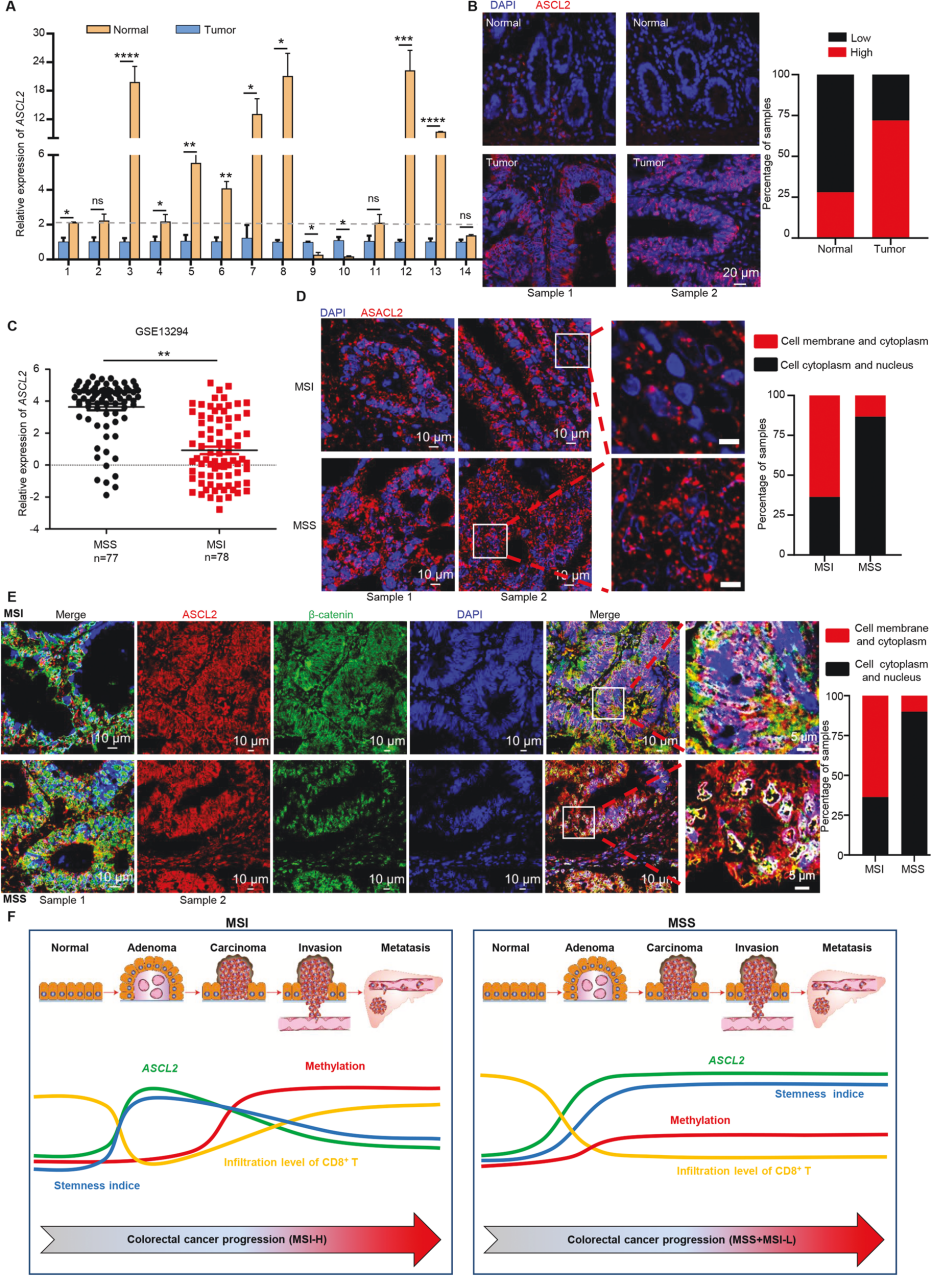
There are two distinct expression patterns of *ASCL2* in MSS and MSI-H CRCs. **A** Expression of *ASCL2* mRNA in the 14 pairs of CRC specimens. Error bars, mean ± SD; *p < 0.05, **p < 0.01, ***p < 0.001, ****p < 0.0001, NS no significance. **B** Representative IF images show low or high levels of anti-ASCL2 staining in CRC tissues and adjacent epithelium. Histograms represent the statistics of ASCL2 expression. Scale bar, 20 μm. **C** Expression of *ASCL2* mRNA in the GSE13294 dataset (77 MSS CRC tumors and 78 MSI CRC tumors). **p < 0.01. **D** Representative immunofluorescence (IF) images indicating ASCL2 protein expression in 11 cases of MSI and 30 cases of MSS CRCs. Histograms represent statistics of major expression locations of ASCL2 in colorectal cancer cells. Scale bar, 10 and 5 μm (right). **E** Representative images of multiplexed immunofluorescence (mIF) showed the expression of ASCL2 and β-catenin in 10 cases of MSI and 10 cases of MSS CRCs. Green = β-catenin, red = ASCL2, blue = DAPI. Histogram represents statistics of major expression locations of ASCL2 and β-catenin in colorectal cancer cells. Scale bar, 10 and 5 μm (right). **F** ASCL2 expression pattern diagram. There are two distinct expression patterns of ASCL2 in MSS and MSI CRCs. The expression of *ASCL2* is silenced by methylation in MSI CRCs, accompanied by decreased stemness index and increased abundance of CD8^+^ T cells infiltrating the tumor. In contrast, in MSS CRCs, the continuous activation of the Wnt signaling pathway leads to increased ASCL2 expression followed by a subsequently increased stemness index, resulting in decreased tumor-infiltrating CD8^+^ T cells.

The Wnt target gene ASCL2 was prominently higher in tumors than in adjacent normal tissues through analyzing both TCGA (The Cancer Genome Atlas) and GEO databases (Supplementary Fig. 1A, B), suggesting a role for abnormal ASCL2 expression in the etiology of colorectal tumorigenesis. Similarly, RT-PCR was used to examine *ASCL2* expression in 14 paired primary tumors and adjacent normal intestinal mucosa, and the results showed significantly higher level of *ASCL2* mRNA in the tumor tissues than in the normal tissues (Fig. 1A). *ASCL2* expression was upregulated in 71.95% (59/82) of the CRC tissues compared to the expression in their matched adjacent normal tissues (Fig. 1B). Surprisingly, there was no significant difference and no obvious trend distribution in the expression level of ASCL2 between different clinical stages in CRC (Supplementary Fig. 1C, D). Moreover, ASCL2 overexpression does not have an impact on the overall or recurrence-free survival rates in CRC patients [9] (Supplementary Fig. 1E), which has led to controversy over the role of *ASCL2* in CRC.

CRCs are highly heterogeneous at the genetic and molecular levels; 15% of CRCs exhibit microsatellite instability (MSI), a molecular indicator of defective DNA mismatch repair (MMR), but the majority are microsatellite-stable (MSS) [31]. Hence, we explored whether the controversial expression of *ASCL2* was related to the phenotype of CRCs. Indeed, these databases revealed that A*SCL2* was more highly upregulated in MSS CRCs than in MSI CRCs (Fig. 1C, Supplementary Fig. 1F, G). Immunofluorescence was used to examine *ASCL2* expression in 11 MSI CRCs and 30 MSS CRCs. And we found that ASCL2 in MSS CRCs was expressed mainly (86.7%) in the nucleus and cytoplasm, while it was mainly expressed (63.7%) in the cytomembrane and cytoplasm in MSI CRCs (Fig. 1D). In despite of which the literature indicates that *ASCL2* is silenced by CpG island methylation during the progression of tumorigenesis, its re-expression is associated with reduced tumor growth [14]. TCGA analysis revealed that the expression of *ASCL2* was negatively correlated with methylation only in MSI-H CRCs (Supplementary Fig. 2A). Moreover, activation of the Wnt signaling pathway is mainly observed in MSS CRCs, suggesting that the upregulation of *ASCL2* expression is related to the activation of Wnt signaling (Supplementary Fig. 2B). Consequently, we examined the co-localization expression of ASCL2 and CTNNB1 in 10 MSI CRCs and 10 MSS CRCs, and results revealed that majority (90%) of MSS CRCs were co-positive primarily in the nucleus and cytoplasm, with activation of the Wnt signaling pathway (Fig. 1E). In contrast, ASCL2 and CTNNB1 were mainly identified in the cell membrane and cytoplasm in 63.7% of MSI CRCs (Fig. 1E). These data suggest that there are distinct expression patterns of *ASCL2* in pMMR/MSS and dMMR/MSI-H CRCs, which helps clarify the controversy of *ASCL2* in CRC research (Fig. 1F).

### Exogenous overexpression of *ASCL2* maintains the stemness phenotype but does not promote the proliferation of CRC cells

**Fig. 2:**
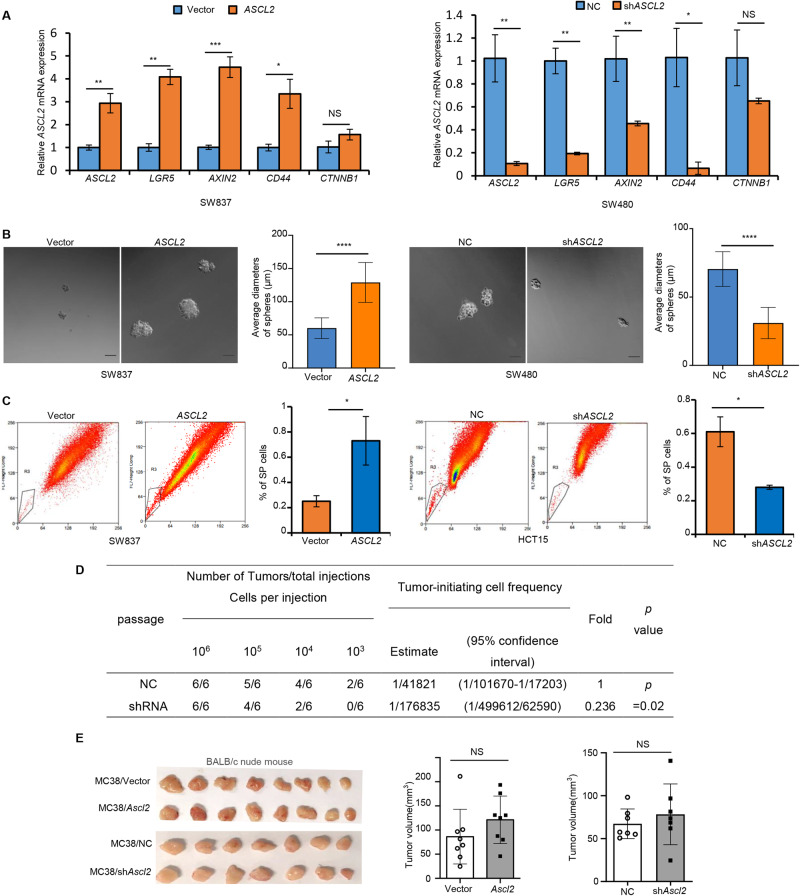
*ASCL2* expression maintains the stemness phenotype without affecting the proliferation of CRC cells. **A** The mRNA levels of stem cell markers (*AXIN2*, *LGR5*, and *CD44*) and *CTNNB1* were examined by RT‒PCR in CRC cells. **B** Single cells were cultured in stem cell medium for 14 days, and the diameters of at least 20 tumorospheres were counted. Histograms represent the statistics of tumorspheres average diameters. Scale bar, 50 μm. **C** SP cells in CRC cells were sorted by flow cytometry following Hoechst 33342 staining. Live tumor cells are shown separated by their emission of Hoechst blue (450/50 nm bandpass filter) and Hoechst red (670/30 nm bandpass filter). Histograms represent the statistics of the percentage of SP cells. **D** The frequencies of tumor-initiating cells were analyzed by extreme limiting dilution assays (ELDAs) in NOD/SCID mice (*n* = 6 mice per group). **E** MC38/Vector cells, MC38/*Ascl2*, MC38/NC and MC38/sh*Ascl2* cells were injected subcutaneously into nude mice (*n* = 8, 8, 7 and 7 mice, respectively). Tumors were collected and measured after the mice were sacrificed. Histograms represent the statistics of tumor volume. All quantification analyses were shown as mean ± SD. *p < 0.05, **p < 0.01, ***p < 0.001, ****p < 0.0001, NS no significance.

As a pivotal determinant of the intestinal stem cell state, we thus investigated the cellular properties of *ASCL2* in CRC cells. TCGA datasets suggested that methylation of *ASCL2* was negatively correlated with stemness in CRCs (Supplementary Fig. 3A), and the expression of ASCL2 was positively correlated with stemness only in pMMR/MSS CRCs (Supplementary Fig. 3B). *ASCL2* overexpression led to a dramatic increase in the expression of stem cell markers, including *LGR5*, *AXIN2*, and *CD44*. The knockdown of *ASCL2* decreased the expression of these markers (Fig. 2A). There was no notable change in *CTNNB1* under the condition of abnormal ASCL2 expression since *ASCL2* is a Wnt/β-catenin target gene[6] (Fig. 2A). When grown in stem cell medium, the overexpression or knockdown of *ASCL2* increased or decreased the propensity of the cells to grow as tumorspheres (Fig. 2B). Consistently, Hoechst side population (SP) analysis revealed that high expression of ASCL2 resulted in elevated numbers of positive cells, and knockdown of ASCL2 resulted in the opposite effect (Fig. 2C). Moreover, limiting dilution xenotransplantation assays demonstrated that knockdown of ASCL2 resulted in a lower tumor-initiating frequency and self-renewing capacity (Fig. 2D), suggesting that *ASCL2* is required for self-renewal. These results demonstrated that *ASCL2* is essential for maintaining stemness in CRC cells. However, subcutaneous xenograft tumors of T cell-deficient nude mice derived by cells with abnormal *ASCL2* expression showed no change in growth kinetics (Fig. 2E). The orthotopic implantation assay demonstrated identical results (Supplementary Fig. 3C). These results suggested that overexpression of *ASCL2* in CRC cells could also be confirmed their ability to self-renew, and their proliferative ability does not change, consistent with the fact that *LGR5*^+^ cancer stem cells depletion did not lead to tumor regression [32].

### Exogenous overexpression of *ASCL2* induces an immune-excluded microenvironment in CRCs in vivo

**Fig. 3:**
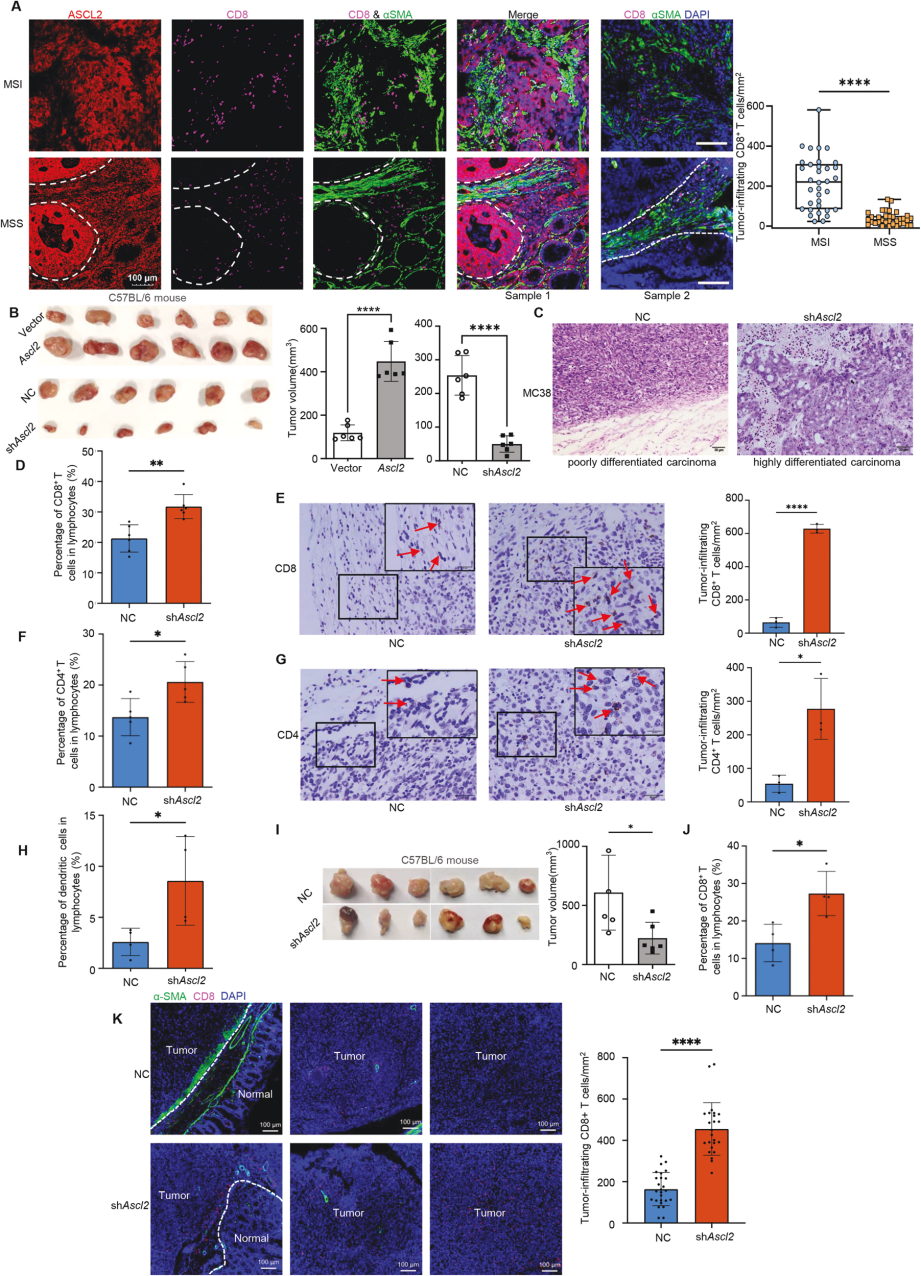
*ASCL2* induces an immune excluded microenvironment in CRCs in vivo. **A** Representative images of mIF indicated the expression of ASCL2, CD8a, and α-SMA in MSI (*n* = 10) and MSS (*n* = 10) CRCs. Green = α-SMA, red = ASCL2, purple = CD8a. Scale bar, 100 μm. Box plot represents the quantification of tumor-infiltrating CD8^+^ T cells density in MSI (*n* = 10) and MSS (*n* = 10) tumors (multiple areas) at 10x objective lens. The middle bar, median; box, inter-quartile range; ****p < 0.0001. **B** MC38/Vector cells, MC38/*ASCL2*, MC38/NC and MC38/sh*ASCL2* cells were injected subcutaneously into injecting C57BL/6 mice (*n* = 6). Histograms represent the statistics of tumor volume. Error bars, mean ± SD; ****p < 0.0001. **C** Subcutaneous tumors formed by MC38/NC and MC38/sh*Ascl2* cells were taken to H&E staining. Scale bar, 50 μm (right). Histograms of tumor-infiltrated CD8^+^ T cells (**D**), CD4^+^ T cells (**F**), and CD11C^+^CD11B^−^ DCs (**H**) cells profiling using flow cytometry, immunohistochemistry (IHC) staining of CD8^+^ T cells (**E**) and CD4^+^ T cells (**G**) in a subcutaneous xenograft tumor model formed by MC38/NC and MC38/sh*Ascl2* cells. The arrows indicate representative monitored immune cells. Histograms represent the quantification of tumor-infiltrating CD8^+^ T cells (**E**) and CD4^+^ T cells (**G**) density in mice tumors (multiple areas) at 20x objective lens. Scale bar, 20 and 10 μm (inset). Error bars, mean ± SD; *p < 0.05, **p < 0.01, ***p < 0.001, ****p < 0.0001. **I** Representative images of primary tumors in C57BL/6 mice (*n* = 6) formed by MC38/NC and MC38/sh*Ascl2* cells in the orthotropic transplantation assay of the intestines. Histogram represents the statistics of tumor volume. Error bars, mean ± SD; *p < 0.05. **J** Histogram represents the statistics of percentage of CD8^+^ T cells in tumor-infiltrating lymphocytes using flow cytometry in the above orthotopic implantation mouse model formed by MC38/NC and MC38/sh*Ascl2* cells. Error bars, mean ± SD; *p < 0.05. **K** Representative images of mIF for CD8 (red) and α-SMA (green) in orthotopic implantation tumor tissue. Scale bar, 100 and 25 μm (inset). Histogram represents the quantification of tumor-infiltrating CD8^+^ T cells density in the orthotopic implantation mouse model (multiple areas) at 10x objective lens. Error bars, mean ± SD; ****p < 0.0001.

MSS CRCs were characterized as poorly infiltrated by effector T cells, whereas MSI tumors showed the opposite characteristic [33]. Therefore, it is crucial to uncover the close relationship between *ASCL2* and the immune microenvironment in CRCs. TIMER2.0 results showed that low ASCL2 expression was significantly associated with six immune cell types (B cells, CD4^+^ T cells, CD8^+^ T cells, macrophages, neutrophils, and dendritic cells) in CRCs (Supplementary Fig. 4A). In addition, we found that MSS CRCs exhibited an immune excluded phenotype in which CD8^+^ T cells were largely excluded from adjacent cancer tissues by CAFs (Fig. 3A), consistent with previous findings [23]; But surprisingly, the situation was different in MSI CRCs. CD8^+^ T cells extensively infiltrated the stroma of normal mucosa and invasive cancer cells in MSI CRCs and were unaffected by CAFs exclusion (Fig. 3A). These results prompted us to examine the impact of *ASCL2* on the immune control of tumor growth in vivo. Our experiments showed that the downregulation of *Ascl2* caused striking tumor growth inhibition and increased the differentiation of cancer cells in MC38 colon tumor models in C57BL/6 mice (Fig. 3B, C). Immunotyping of single cells isolated from tumor tissues and immunohistochemistry (IHC) analysis revealed that *Ascl2* markedly decreased the number of tumor-infiltrating CD8^+^ T cells, CD4^+^ T cells, and CD11C^+^ CD11B^-^ DCs (Fig. 3D–H and Supplementary Fig. 4B–D), suggesting immune activation in the TME. Moreover, *Ascl2* had no apparent effect on the tumor growth of MC38 tumors established in T cell-deficient nude mice (Fig. 2E), suggesting the critical role of T cells in the *Ascl2*-induced antitumor effect. Orthotopic implantation assays demonstrated that knockdown of *Ascl2* suppressed the growth of primary CRC tumors in C57BL/6 mice instead of in nude mice (Fig. 3I and Supplementary Fig. 3C). Consistent with the subcutaneous tumorigenesis assay in C57BL/6 mice, downregulation of *Ascl2* observably increased the number of tumor-infiltrating CD8^+^ T cells in primary CRC tumors (Fig. 3J and Supplementary Fig. 4E). As shown in Supplementary Fig. 4F, G, ectopic expression of *Ascl2* had no influence on liver metastases in the two colon tumor models. Furthermore, we found that *Ascl2* contributed to excluding CD8^+^ T cells from tumor parenchyma through activated CAFs. In control mice, large numbers of CD8^+^ T cells were distributed in the normal mucosa around tumor, resulting a poor tumor-infiltrating CD8^+^ T cells; On the contrary, knockdown of *ASCL2* in tumor cells resulted in fewer activated CAFs followed by reduced infiltration of CD8^+^ T cells, and without obvious effect on CAFs proliferation (Fig. 3K and Supplementary Fig. 4H). Thus, we assumed that *ASCL2* induced the excluded microenvironment by decreasing CD8^+^ T cell infiltration in CRCs.

### Deletion of intestinal epithelial *Ascl2* promotes inflamed immune microenvironment formation in conditional knockout mice

**Fig. 4:**
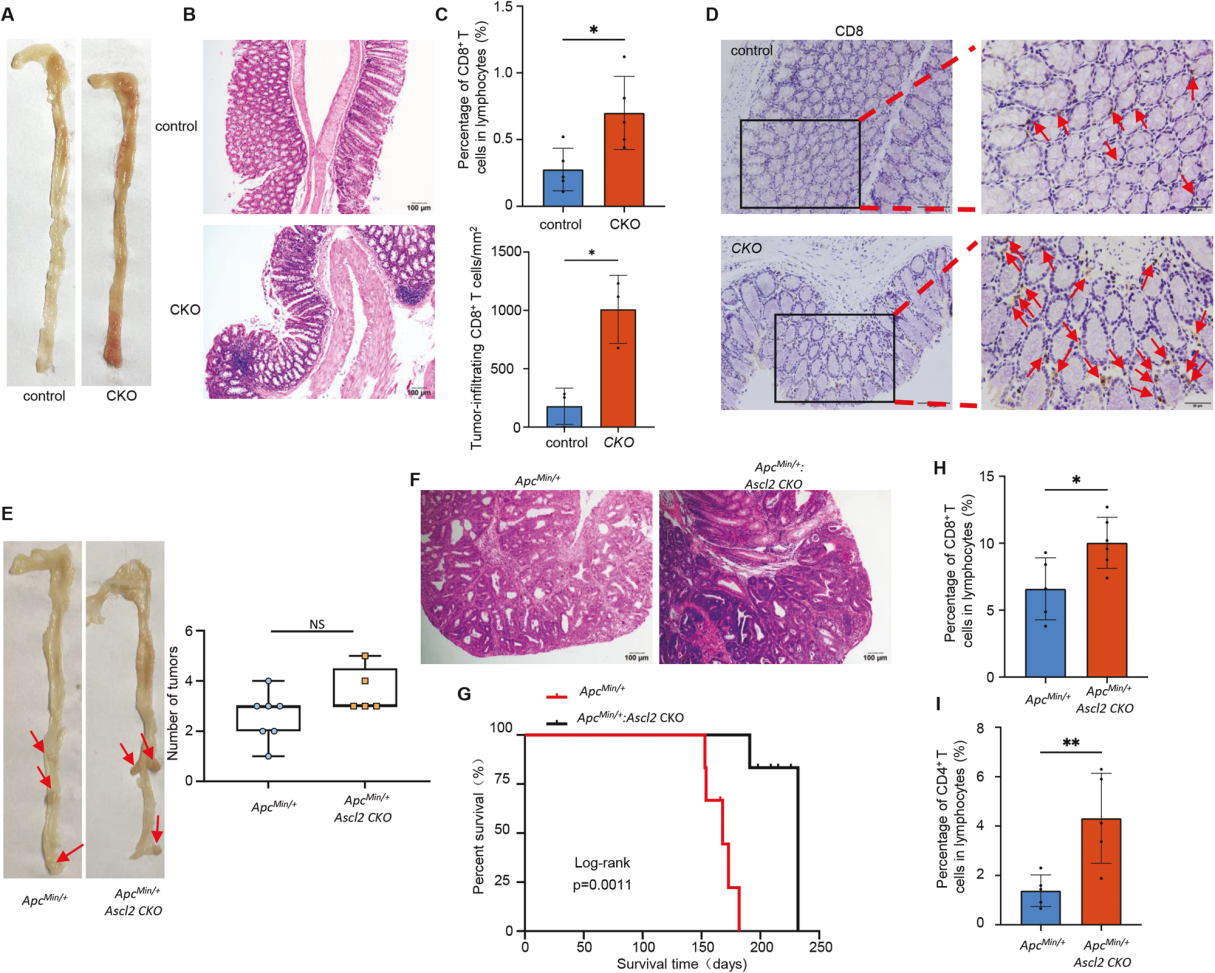
ASCL2 CKO mice show enhanced inflamed immune microenvironment formation. Representative images of macroscopic polyps (**A**) and H&E staining (**B**) in the *Ascl2* CKO mice and control (*Ascl2*^*flox/flox*^) mice. Scale bar, 100 μm. **C** Histogram of infiltrating CD8^+^ T cells in the intestinal epithelium as assayed by flow cytometry in the *Ascl2* CKO mice and control (*Ascl2*^*flox/flox*^) mice. Error bars, mean ± SD; *p < 0.05. **D** IHC staining of CD8^+^ T cells in the intestinal epithelium of the *Ascl2* CKO mice and control mice. The arrow indicates representative monitored immune cells. Histogram represents the quantification of infiltrating CD8^+^ T cells in the intestinal epithelium of the CKO mice and control mice at 20x objective lens. Scale bar, 50 and 20 μm (right). Representative images of macroscopic polyps (**E**) and H&E staining (**F**) in the *Apc*^*Min/+*^ mice and *Apc*^*Min/+*^_;_ *Ascl2* CKO mice (n = 7 and 5 mice, respectively), as well as monitoring overall survival (*n* = 6) (**G**), determined by the Kaplan–Meier analysis (*p* < 0.05). Box plot represents the statistics of tumor numbers. Scale bar, 100 μm. The middle bar, median; box, inter-quartile range; NS no significance. Histograms of infiltrating CD8^+^ T cells (**H**) and CD4^+^ T (**I**) cells in the tumor tissue as assayed by flow cytometry in the *Apc*^*Min/+*^ mice and *Apc*^*Min/+*^_;_ *Ascl2* CKO mice. Error bars, mean ± SD; *p < 0.05.

To determine whether *Ascl2* affects the components of the microenvironment in intestinal epithelial cells, we generated an intestinal epithelium-specific *Ascl2* conditional knockout mouse strain (*Ascl2* CKO) (Supplementary Fig. 5A). And the result showed that the expression of ASCL2 in *Ascl2* CKO mice intestinal epithelial tissues was significantly weaker than that in control mice by immunofluorescence (Supplementary Fig. 5B). There were no significant differences in survival between the two cohorts of mice (data not shown). However, macroscopic observation showed that colorectal mucosal inflammation became aggravated in the CKO mice (Fig. 4A), which was confirmed by histological analysis (Fig. 4B). Furthermore, we validated the proportion of CD8^+^ T cells in transgenic mouse colonic intestines. Similar results were obtained in which knockout of *Ascl2* increased the number of CD8^+^ T cells (Fig. 4C, D and Supplementary Fig. 5C).

We next investigated the consequence of the absence of *Ascl2* expression in intestinal tumorigenesis. Hence, we crossed the *Ascl2* CKO mice with the *Apc*^*Min/+*^ model, generating *Apc*^*Min/+*^_;_ *Ascl2* CKO mice (Supplementary Fig. 5A). Although no significant differences in tumor burden were found between the cohorts of *Apc*^*Min/+*^and *Apc*^*Min/+*^_;_ *Ascl2* CKO mice (Fig. 4E, F), the survival of the *Apc*^*Min/+*^_;_ *Ascl2* CKO mice were surprisingly longer than that of the control mice (Fig. 4G). Furthermore, we observed significantly enhanced infiltration of CD8^+^ T cells and CD4^+^ T cells in the *Apc*^*Min/+*^_;_ *Ascl2* CKO mice (Fig. 4H, I and Supplementary Fig. 5D, E). Similarly, a certain number of CAFs was observed within cohorts of *Apc*^*Min/+*^_;_ *Ascl2* CKO tumors, although CAFs proliferation had little effect (Supplementary Fig. 5F). Taken together, these transgenic mouse results suggested that deletion of intestinal epithelial *Ascl2* could accelerate the distribution of CD8^+^ T cells in the intestinal microenvironment of mice.

### *Ascl2* promotes CRCs progression by inhibiting CD8^+^ T cell infiltration in the tumor microenvironment

**Fig. 5:**
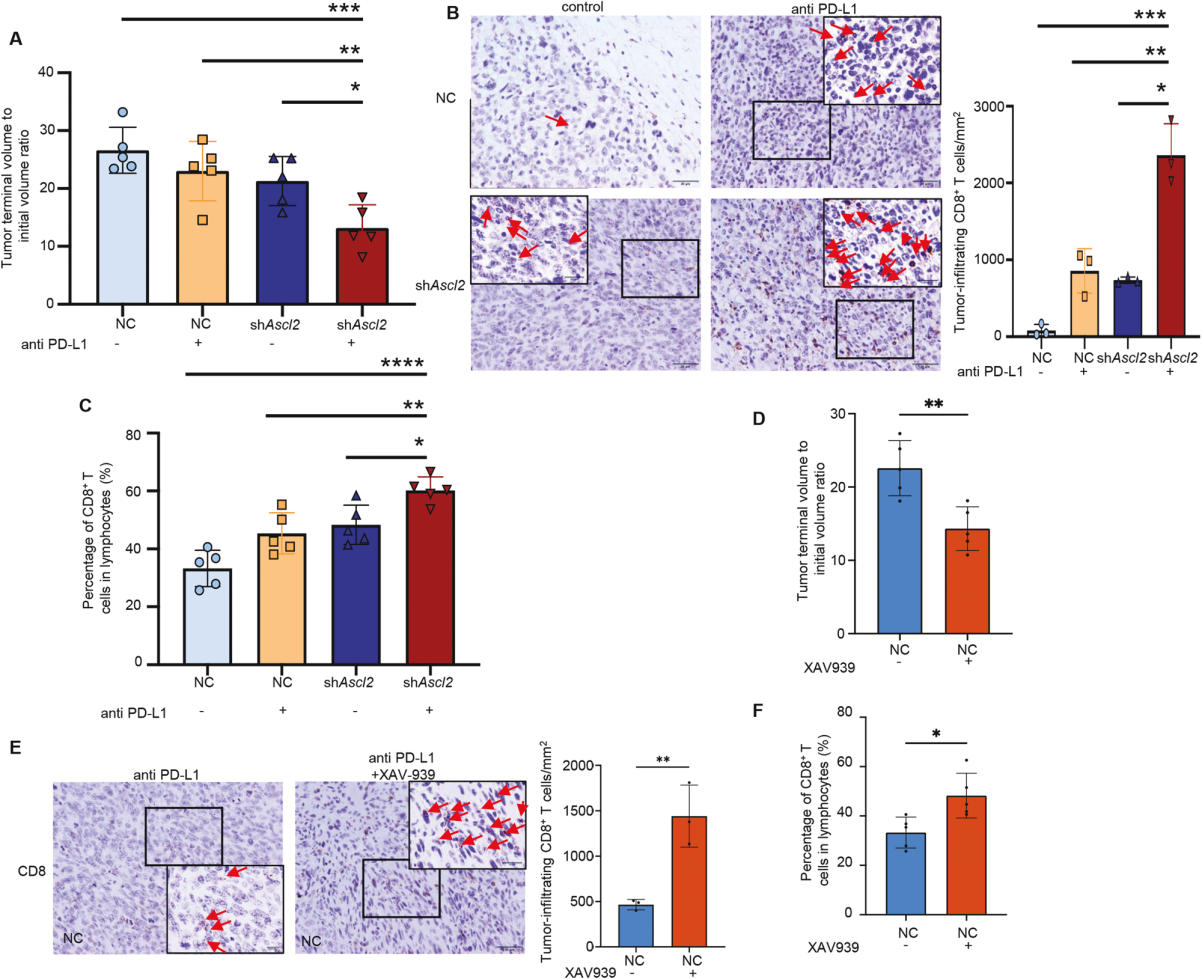
*Ascl2* promotes CRCs progression by inhibiting CD8^+^ T cell infiltration in the tumor microenvironment. **A** The ratio of the tumor terminal volume to the initial volume after anti-PD-L1 treatment in the mouse CRC model formed by MC38/NC and MC38/sh*Ascl2* cells. Error bars, mean ± SD; **p < 0.01, ***p < 0.001. **B** Representative images of IHC for CD8^+^ T cells in the mouse tumor treated with anti-PD-L1. Scale bar, 20 and 10 μm (inset). The arrows indicate representative monitored immune cells. Histogram represents the quantification of tumor-infiltrating CD8^+^ T cells in the mice (*n* = 3) at 20x objective lens. Error bars, mean ± SD; *p < 0.05, **p < 0.01. **C** Tumor-infiltrated CD8^+^ T cell profiling in the mouse CRC model treated with anti-PD-L1 by flow cytometry. Histogram represents the statistics of the percentage of CD8^+^ T cell in tumor-infiltrating lymphocytes. Error bars, mean ± SD; *p < 0.05, **p < 0.01, ****p < 0.0001. **D** The ratio of the tumor terminal volume to the initial volume with or without XAV939 treatment in the mouse CRC model formed by MC38/NC and MC38/sh*Ascl2* cells. Error bars, mean ± SD; *p < 0.05. **E** Representative images of IHC for CD8^+^ T cells in the mouse tumor treated with XAV939. Scale bar, 20 and 10 μm (inset). Histogram represents the quantification of tumor-infiltrating CD8^+^ T cells in the mice (*n* = 3) at 20x objective lens. Error bars, mean ± SD; **p < 0.01. **F** Tumor-infiltrated CD8^+^ T cell profiling in the mouse CRC model treated with XAV939 by flow cytometry. Histogram represents the statistics of the percentage of CD8^+^ T cell in tumor-infiltrating lymphocytes. Error bars, mean ± SD; *p < 0.05.

Higher CD8^+^ T cell density was associated with a higher objective response rate and duration of disease control to anti-PD-L1 antibody [34]. Conversely, the low density of T-cells may indicate an inadequate response to ICIs. To test our hypothesis that reduced CD8^+^ T cell infiltration by *ASCL2* limits the response to anti-PD-L1 in CRCs, we studied the MC38 mouse colorectal cancer model. Mice with established subcutaneous MC38/NC or MC38/sh*Ascl2* tumor cells were treated with anti-PD-L1 antibody. As expected, anti-PD-L1 did not have a significant therapeutic effect on MC38/NC tumor growth, but the growth rate of MC38/sh*Ascl2* mice treated with antibodies against PD-L1 was significantly slowed down (Supplementary Fig. 6A), and the tumor burden was significantly reduced (Fig. 5A). The MC38/sh*Ascl2* mice with antibody blockade also showed a substantial increase in tumor-infiltrating CD8^+^ T cells (Fig. 5B, C and Supplementary Fig. 6B). Consistently, further administration of the Wnt signaling inhibitor XAV-939 combined with anti-PD-L1 treatment partly enhanced the therapeutic effect of anti-PD-L1 in MC38 tumors (Fig. 5D). IHC and flow cytometric results demonstrated that the combined use of XAV-939 significantly elevated the proportion of CD8^+^ T cells (Fig. 5E, F, and Supplementary Fig. 6C). Together, these results suggested that *Ascl2* inhibition potentiated the ability of anti-PD-L1 to enhance antitumor immunity, resulting in optimal CD8^+^ T cell positioning and ensuing tumor regression.

### *ASCL2* induces CAFs activation to exclude CD8^+^ T cells by transcriptionally activating *TGFB*

**Fig. 6:**
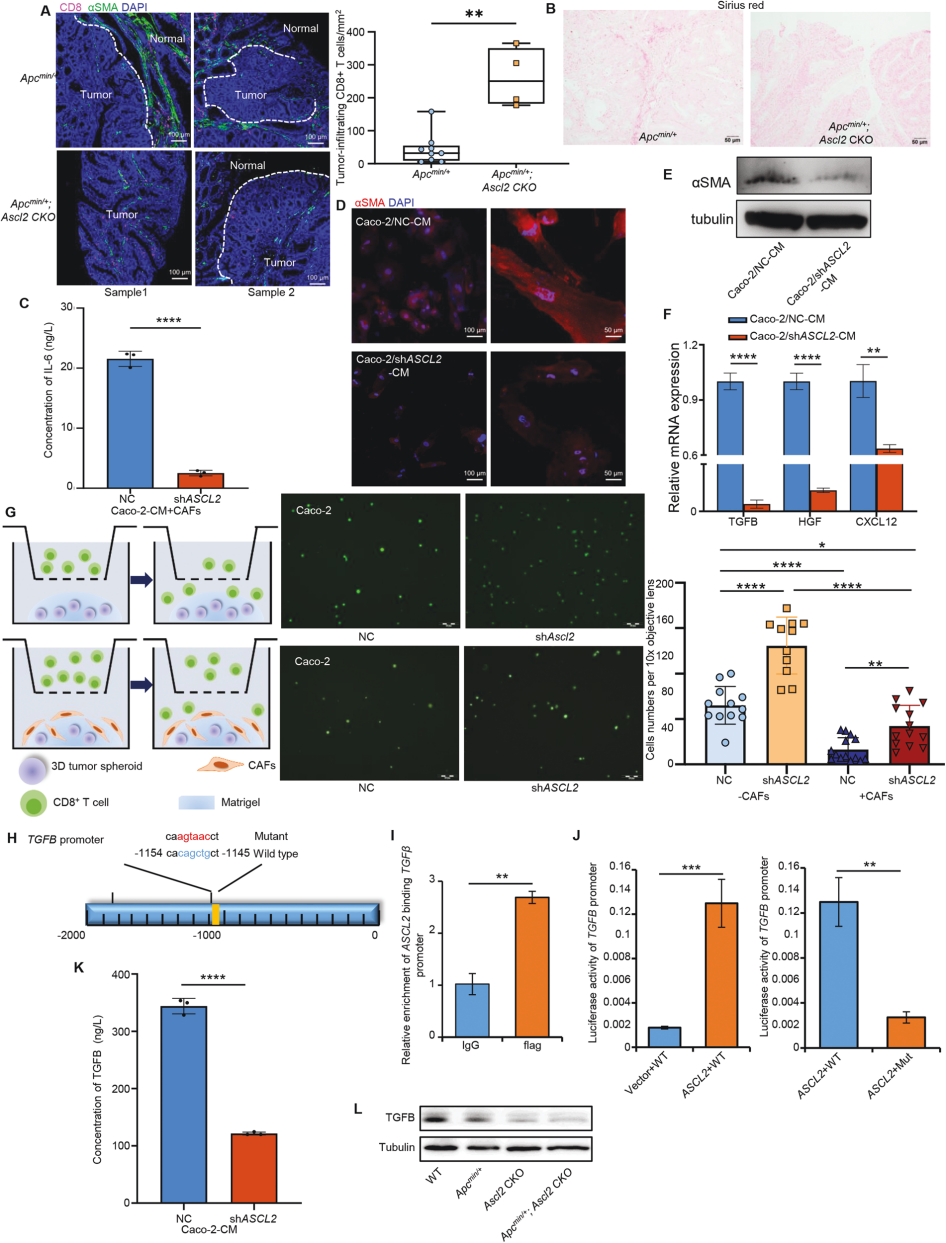
*ASCL2* induces CAFs activation to exclude CD8^+^ T cells by transcriptionally activating *TGFB*. **A** Representative images of mIF for CD8 (purple) and α-SMA (green) in the *Apc*^*Min/+*^ mice and *Apc*^*Min/+*^_;_ *Ascl2* CKO mice tumor. Scale bar, 100 μm. Histogram represents the quantification of tumor-infiltrating CD8^+^ T cells density in mice tumors (multiple areas from 3 tumors) at 10x objective lens. The middle bar, median; box, inter-quartile range; ***p < 0.001. **B** Collagen deposition using Sirius red staining in the *Apc*^*Min/+*^ mice and *Apc*^*Min/+*^_;_ *Ascl2* CKO mice tumor. Scale bar, 50 μm. **C**–**F** Caco-2 cells were transfected with sh*ASCL2*, and the supernatant was filtered and collected after 48 h, followed by coculture with human primary CAFs for 48 h. IL-6 production was measured by ELISA (**C**). The expression of αSMA (red) was detected by IF (**D**) and Western blot (**E**). The relative mRNA expressions of functional molecules CXCL12, TGFB, and HGF were detected by RT‒PCR (**F**). Error bars, mean ± SD; **p < 0.001. ****p < 0.0001. Scale bar, 100 and 50 μm. **G** CD8^+^ T cell migration assay diagram showed activated CD8^+^ T cells labeled with CFSE were indirectly cocultured with preformed 3D tumor spheroid (left and top). Based on the migration assay model, preformed 3D tumor spheroid was co-cultured with human primary CAFs (left and bottom), followed by activated CD8^+^ T cells labeled CSFE placed in the upper chamber and visualized by fluorescence microscopy (middle). Histogram represents the statistics of CD8^+^ T cells in the lower chamber (right). Scale bar, 50 μm. Error bars, mean ± SD; *p < 0.05, **p < 0.01, ****p < 0.001. **H** Schematic diagram of the *TGFB* promoter, the position of *ASCL2* binding sites is indicated by yellow rectangles, and the mutated nucleotides of *ASCL2* binding motif in *TGFB* promoter are highlighted in red. **I** ChIP was performed to analyze *ASCL2* binding to the *TGFB* promoter. RT‒PCR experiment was performed with primers against the indicated area in the *TGFB* promoter. Error bars, mean ± SD; **p < 0.01. **J** Relative expression of WT or mutant *TGFB* promoter-driven luciferase reporters in *ASCL2*-overexpressing cells. Error bars, mean ± SD; **p < 0.01, ***p < 0.001. **K** Caco-2 cells were transfected with sh*ASCL2* for 48 h before *TGFB* was assayed using ELISA. Error bars, mean ± SD; **p < 0.01. **L** Protein expression of TGFB in intestinal epithelial cells in indicated mice. α-Tubulin was used as a loading control.

Compared with the *Apc*^*Min/+*^_;_ *Ascl2* CKO mice, *Apc*^*Min/+*^ mice displayed more activated CAFs and reduced tumor-infiltrating CD8^+^ T cells, leading to a tumor suppressive immune microenvironment (Fig. 6A). Therefore, we hypothesized that *ASCL2* plays a central role in affecting CAFs activation. Indeed, knocking out *Ascl2* in intestinal epithelial cells significantly reduced collagen deposition (Fig. 6B). Continuous crosstalk between cancer cells and CAFs enhances tumor growth and invasion [35]. To determine the ASCL2 functional effect on the CAFs, we derived primary CAFs from CRC patients and used the culture supernate from CRC cells to culture CAFs. Downregulation of ASCL2 in CRC cells suppressed IL-6, which prevents T cell recruitment into the TME [36], released by CAFs (Fig. 6C and Supplementary Fig. 7A). Notably, the supernatant culture of *ASCL2* knockdown CRC cells reduced CAFs phenotype markers characterized by a-SMA expression (Fig. 6D, E). Similar results were obtained in the mRNA expression of genes associated with the activation and functionality of CAFs. The expressions of functional molecules TGFB, HGF and CXCL12 were dramatically decreased (Fig. 6F). Furthermore, we examined the role of CAFs around the tumor through CD8^+^ T cell migration assay. Activated CD8^+^ T cells migrated from the upper compartment when they were co-cultured with 3D tumor spheres, while the CD8^+^ T cells cocultured with ASCL2-downregulated 3D tumor spheroid had a stronger migration (Fig. 6G). Simultaneously, when human CAFs seeded on the top of Matrigel containing 3D tumor spheres, activated CD8^+^ T cells were intercepted by the CAFs barrier which had a potential immunosuppressive effect, and resulted in an obviously reductive CD8^+^ T cell migration, even when CAFs were co-cultured with ASCL2-downregulated 3D tumor spheroid (Fig. 6G). These results suggest that *ASCL2* activates CAFs and may contribute to the immune suppressive TME.

The recruitment of activated fibroblasts is dependent on TGFB signaling, which regulates a myriad of mainly immunosuppressive responses [37]. GTRD (Gene Transcription Regulation Database) was used to analyze the possible transcription target genes of *ASCL2*, and the positively related genes in CRCs tissues were assessed. The results showed that the inflammatory inhibitors *TGFB* might be regulated by *ASCL2* (Supplementary Fig. 7B). Therefore, we analyzed the promoter sequence of the *TGFB* gene and detected the sequence of the binding site for *ASCL2* (Fig. 6H). Furthermore, we performed chromatin immunoprecipitation (ChIP) assays, and the results revealed that the ASCL2 protein could bind to the *TGFB* promoter at the candidate site (Fig. 6I). Moreover, the results of the dual luciferase reporter assay revealed that *ASCL2* activated the wild-type *TGFB* promoter but not the mutant promoter (Fig. 6J).

Then we also detected reduced TGFB release in the supernatant of the *ASCL2*-downregulated cells (Fig. 6K and Supplementary Fig. 7C). Besides, *ASCL2* negatively regulated NF-κB signaling pathway, which was identified as critical regulator of the initiation and resolution of inflammation [38], and shRNA-mediated depletion of *TGFB* further facilitated activation (Supplementary Fig. 7D). In vivo, the expression level of intestinal TGFB was reduced in the *Ascl2* CKO mice (Fig. 6L). The mRNA levels of genes in the TGFβ-Smad signaling pathway which are associated with CAFs activation [39] were also reduced (Supplementary Fig. 7E). Taken together, these results indicated that *ASCL2* plays a crucial role in regulating the intestinal epithelial inflammatory microenvironment by activating CAFs through transcriptional activation of *TGFB*.

## Discussion

In recent years, the role of *ASCL2* in tumorigenesis has become a research hotspot. As a target gene of the Wnt pathway, *ASCL2* is involved in maintaining and renewing intestinal stem cells. However, its expression pattern in CRCs remains controversial. Previous results showed that *ASCL2* was significantly upregulated at all stages of CRCs [9, 40] and that *ASCL2* and 11p15.5 were amplified in CRCs [10]. However, *ASCL2* has also been reported to be silenced by methylation in CRCs [14]. Here, we found two distinct expression patterns of *ASCL2* in pMMR/MSS and dMMR/MSI-H CRC by analyzing CRC datasets in the TCGA and GEO public databases. In pMMR/MSS CRCs, Wnt signaling was aberrantly activated, which resulted in the upregulation of the target gene *ASCL2*. In contrast, most MSI tumors are classified as CMS1, which is one of CMSs, displayed a widespread hypermethylation status, dominated by CpG island methylator phenotype [16]. *ASCL2* was downregulated in a methylation-dependent manner in dMMR/MSI CRCs.

In addition, the role of dysregulation of *ASCL2* expression in the initiation and progression of CRCs remains contradictory [11–13, 15]. Our results showed that exogenous overexpression of *ASCL2* induces an immune-excluded microenvironment in CRCs in vivo; in contrast, deletion of intestinal epithelial *Ascl2* promotes inflamed immune microenvironment formation in conditional knockout mice. These results suggest that the dysregulation of *ASCL2* may promote or inhibit the initiation and progression of CRC by remodeling the tumor immune microenvironment rather than directly stimulating the proliferation and migration of tumor cells. Recently, Wu, L. and Yang, Q. et al. also confirmed that *ASCL2* was involved in the regulation of the immune-excluded microenvironment in pMMR/MSS CRCs [21, 22].

Recent studies have also shown that malignant tumor cells with Wnt pathway activation have strong stemness but relatively few infiltrating immune cells in the tumor microenvironment, which is not sensitive to ICI treatment. However, the regulatory relationship among the stemness of tumor cells, the immune excluded microenvironment and ICI treatment resistance is still unclear [17]. Approximately 90% of CRCs cases display permanent activation of the Wnt signaling pathway [41, 42]. *ASCL2*, a critical transcriptional regulatory target gene, is overexpressed in pMMR/MSS CRCs and maintains a stemness phenotype, accompanied by a lower density of TILs than in dMMR/MSI CRCs. In addition, a WNT pathway inhibitor combined with anti-PD-L1 antibodies facilitated T cell infiltration and provoked strong antitumor immunity and tumor regression in the MC38/sh*Ascl2* mouse CRC model.

Mounting evidence has shown that CAFs define an immune-excluded microenvironment by preventing the infiltration of immune cells into tumors and inhibiting immune cell activation [24, 43–45]. We observed the same result in most CRCs, but surprisingly, the situation was different in MSI CRCs. We found that CD8^+^ T cells extensively infiltrated the stroma of normal mucosa and invasive cancers and were unaffected by CAFs exclusion in MSI CRCs. And further, our results showed that *ASCL2* expression correlates with highly reactive inflammatory desmoplastic stroma, remodeling of the immune-excluded microenvironment, and proliferation of CRC cells. Knockdown of *ASCL2* inhibited the growth and activation of immunosuppressive CAFs. Furthermore, deletion of *Ascl2* eliminated the ability of CAFs to decrease tumor-infiltrating CD8^+^ CTLs in the C57BL/6 J and *Ascl2* CKO mice. Significantly, the immunomodulatory functions of CAFs are realized by the secretory phenotype, enabling the production of large amounts of cytokines and chemokines.

The crosstalk between parenchymal cells and CAFs in many human malignant tumors is dependent on *TGFB* secreted from cancer cells [26]. *TGFB* signaling exerts many mainly immunosuppressive responses by preventing the infiltration of immune cells and suppressing the differentiation and activity of T cells, thus attenuating the intrinsic antitumor potential of immune cells within the TME [37, 46, 47]. Our data show that *ASCL2* a highly reactive inflammatory desmoplastic stroma riched in activated CAFs and remodeled the immune excluded microenvironment by transcriptionally activating *TGFB* in pMMR/MSS CRC cells. Targeting *TGFB* has indeed enhanced tumor susceptibility to anti-PD-1/PD-L1 therapies [28, 48, 49], which promises to be an effective strategy that may have good prospects for the treatment of pMMR/MSS CRCs. The feasibility and efficacy of combination treatment with anti-PD-L1 and TGFB inhibitors require further studies. In conclusion, we investigated the molecular mechanisms underlying immune-excluded microenvironment remodeling and further confirmed that Wnt pathway inhibitors combined with ICIs present a novel strategy and therapeutic opportunity for pMMR/MSS CRCs.

**Fig. 7:**
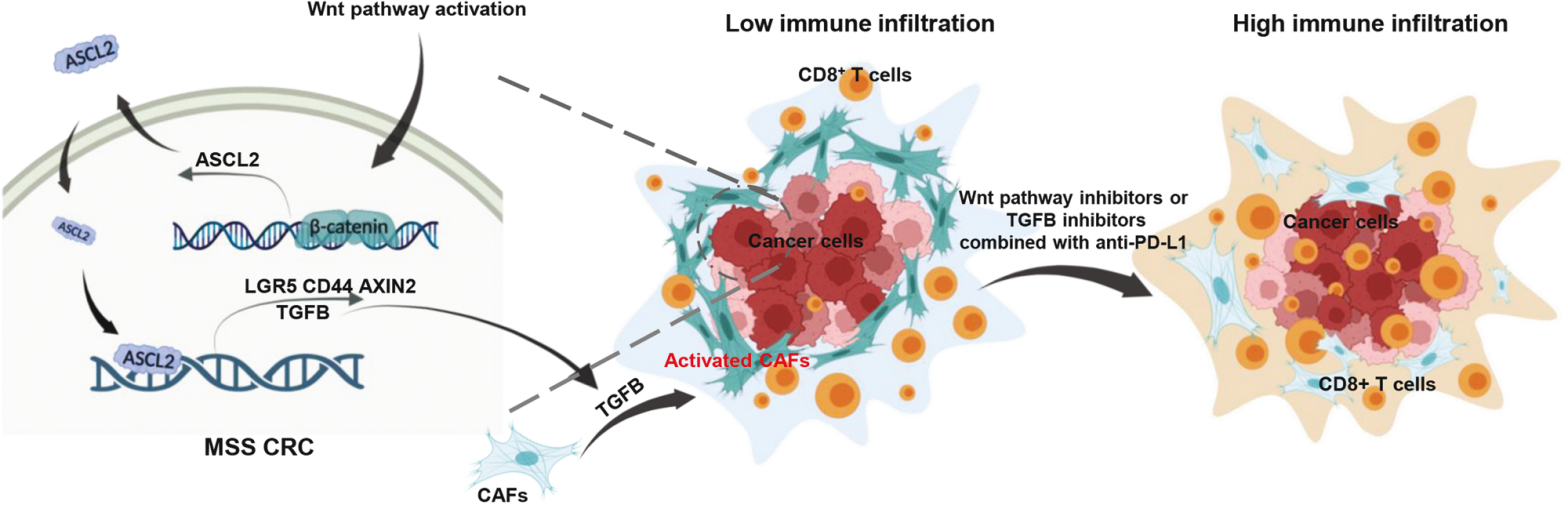
Schematic representation of this study. In MSS CRCs, abnormal activation of Wnt signaling pathway leads to increased expression of target gene ASCL2, resulting in enhanced stemness of CRC cells. Meanwhile, ASCL2 activates CAFs through transcriptional activation of TGFB, and CD8^+^ T cells are blocked from entering tumor cells by CAFs barriers, inducing an immune-excluded microenvironment.

In summary, we uncovered distinct expression patterns of *ASCL2* in pMMR/MSS and dMMR/MSI-H CRCs and to some extent resolved the lingering controversy. Moreover, we showed that *ASCL2* induces an immune-excluded microenvironment by activating CAFs through transcriptional activation of *TGFB* secreted from cancer cells, paving the way for Wnt pathway or TGFB inhibitors combined with ICIs in pMMR/MSS CRCs, which presents a novel strategy and therapeutic opportunity (Fig. 7).

## Supplementary information

supplementary tables

supplementary information

## Data Availability

All the other data supporting the findings of this study are available within the article and its Supplementary Information files and from the corresponding author upon reasonable request.
