# Supplementary material for: ASCL2 induces an immune excluded microenvironment by activating cancer-associated fibroblasts in microsatellite stable colorectal cancer
Source: Oncogene. 2023 Aug 17;42(38):2841–53. doi: 10.1038/s41388-023-02806-3 (PMC10504082; doi:10.1038/s41388-023-02806-3)
Supplement: Supplementary file 1 — supplementary tables [file 41388_2023_2806_MOESM1_ESM.docx]

**Supplementary tables 1 primary antibody**

| **Antibody** | **Vender** | **Cat.No** | **Dilution** |
| --- | --- | --- | --- |
| ASCL2 | Millipore | MAB4418 | 1:100(mIF); 2 µg for 20 µg of  chromatin (ChIP) |
| β-catenin | ProteinTech | 66379-1-Ig | 1:100 (mIF) |
| α-SMA | ProteinTech | 67735-1-Ig | 1:5000 (mIF) |
| α-SMA | ProteinTech | 14395-1-AP | 1:1000 (WB) |
| CD8a | Cell Signaling Technology | 98941 | 1:400 (IHC) |
| CD4 | Cell Signaling Technology | 25229 | 1:200 (IHC) |
| Ki67 | BD biosciences | 550609 | 1:500 (mIF) |
| TGFB | Bioss | bs-0086R | 1:500 (WB) |
| CD8 | ZSGB-BIO | ZA-0508 | working solution (mIF, IF) |
| NF-κB Pathway Antibody Sampler Kit | Cell Signaling Technology | 9936 | 1:000 (WB) |
| Tubulin | Rayantibody | RM2007 | 1:10000 (WB) |

**Supplementary tables 2 Primer Sequences Used for RT-PCR (5' to 3')**

| **Gene** | **Species** | **Sense (5'-3')** | **Antisense (5'-3')** |
| --- | --- | --- | --- |
| *GAPDH* | human | GACTCATGACCACAGTCCATGC | AGAGGCAGGGATGATGTTCT |
| *ASCL2* | human | CGTGAAGCTGGTGAACTTGG | GGATGTACTCCACGGCTGAG |
| *LGR5* | human | CCTGCTTGACTTTGAGGAAGACC | CCAGCCATCAAGCA GGTGTTCA |
| *AXIN2* | human | CAAACTTTCGCCAACCGTGGTTG | GGTGCAAAGACATAGCCAGAACC |
| *CD44* | human | CTGCCGCTTTGCAGGTGTA | CATTGTGGGCAAGGTGCTATT |
| *CTNNB1* | human | AGCTTCCAGACACGCTATCAT | CGGTACAACGAGCTGTTTCTAC |
| *HGF* | Human | GCTATCGGGGTAAAGACCTACA | CGTAGCGTACCTCTGGATTGC |
| *CXCL12* | Human | ATTCTCAACACTCCAAACTGTGC | ACTTTAGCTTCGGGTCAATGC |
| *TGFB* | Human | CTAATGGTGGAAACCCACAACG | TATCGCCAGGAATTGTTGCTG |
| *GAPDH* | mouse | AGGTCGGTGTGAACGGATTTG | GGGGTCGTTGATGGCAACA |
| *Tgfβ* | mouse | CTTCAATACGTCAGACATTCGGG | GTAACGCCAGGAATTGTTGCTA |
| *Timp1* | mouse | GCAACTCGGACCTGGTCATAA | CGGCCCGTGATGAGAAACT |
| *Ctgf* | mouse | GGGCCTCTTCTGCGATTTC | ATCCAGGCAAGTGCATTGGTA |
| *Smad2* | mouse | AAGCCATCACCACTCAGAATTG | CACTGATCTACCGTATTTGCTGT |
| *Tgfbr1* | mouse | TCTGCATTGCACTTATGCTGA | AAAGGGCGATCTAGTGATGGA |
| *Smad1* | mouse | GCTTCGTGAAGGGTTGGGG | CGGATGAAATAGGATTGTGGGG |
| *Smad4* | mouse | ACACCAACAAGTAACGATGCC | GCAAAGGTTTCACTTTCCCCA |
