# Supplementary material for: ASCL2 induces an immune excluded microenvironment by activating cancer-associated fibroblasts in microsatellite stable colorectal cancer
Source: Oncogene. 2023 Aug 17;42(38):2841–53. doi: 10.1038/s41388-023-02806-3 (PMC10504082; doi:10.1038/s41388-023-02806-3)
Supplement: Supplementary file 2 — supplementary information [file 41388_2023_2806_MOESM2_ESM.pdf]

Supplementary figure and figure legends

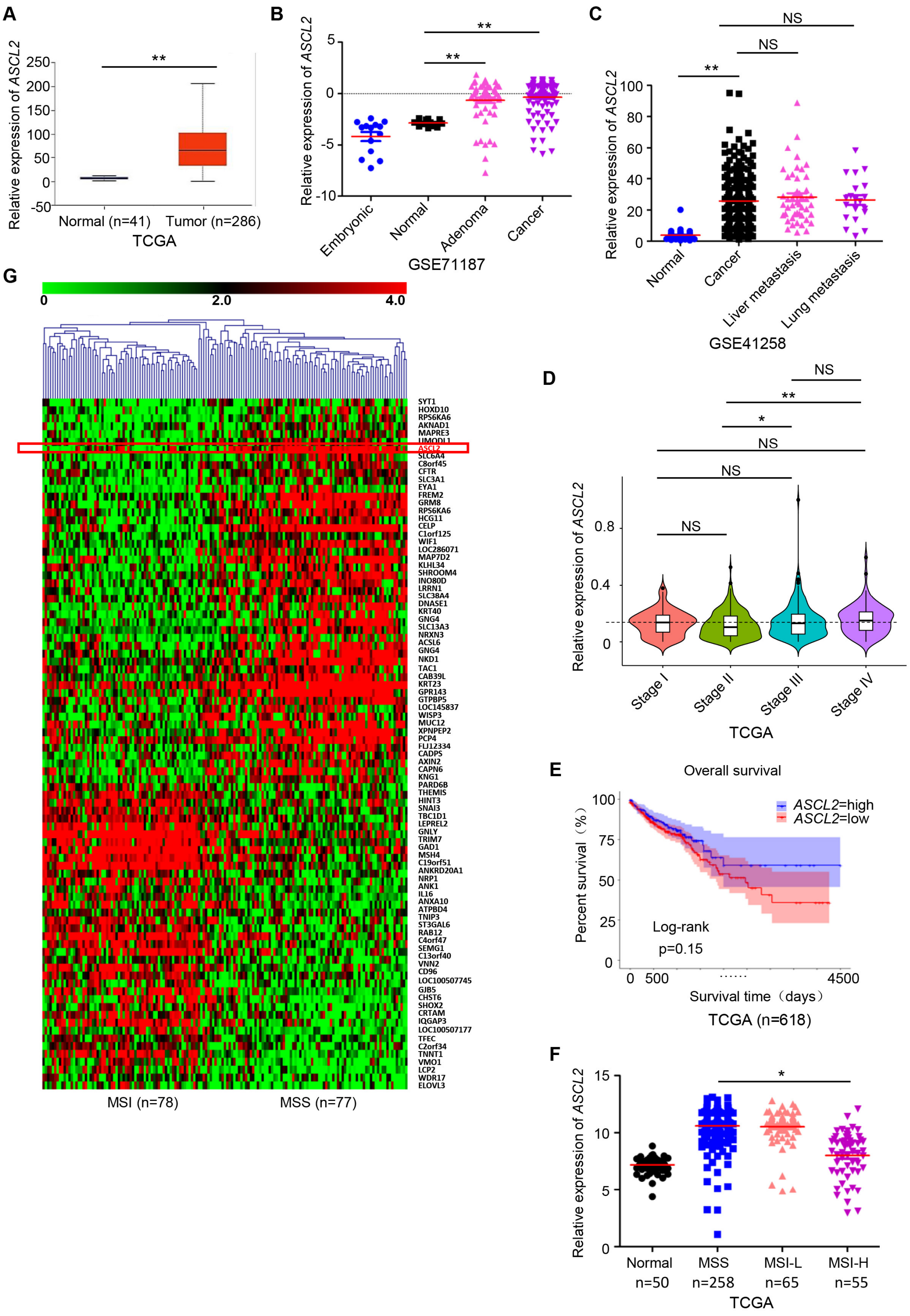

Supplementary Fig. 1 Analysis of *ASCL2* expression patterns in MSS and MSI-H CRCs

(A) Expression of *ASCL2* mRNA in TCGA (The Cancer Genome Atlas) CRCs database (286

tumors and 41 normal tissues). Error bars, mean ± SD; **p<0.01. (B) Expression of *ASCL2* mRNA in intestinal tissues at various stages of development in the GSE71187 dataset. Error bars, mean ± SD; **p<0.01. (C) Expression of *ASCL2* mRNA in normal intestinal epithelial tissues, CRC tissues and metastatic cancer tissues in GSE41258 dataset. Error bars, mean ± SD; **p<0.01; NS, no significance.

(D) Expression of *ASCL2* mRNA in TCGA CRCs database between different clinical stages. **p<0.01; NS, no significance; *p<0.05. (E) Overall survival of CRC patients correlates with ASCL2 expression level in TCGA CRCs database (618 patients). (F) Expression of *ASCL2* mRNA in TCGA CRCs database (50 normal tissues, 258 MSS tumors, 65 MSI-Low tumors and 55 MSI-High tumors). *p<0.05. (G) Heat map represent mRNA expression of multiple genes in MSS CRCs and MSI CRCs in GSE13294 dataset (including 77 MSS CRC tumors and 78 MSI CRC tumors).

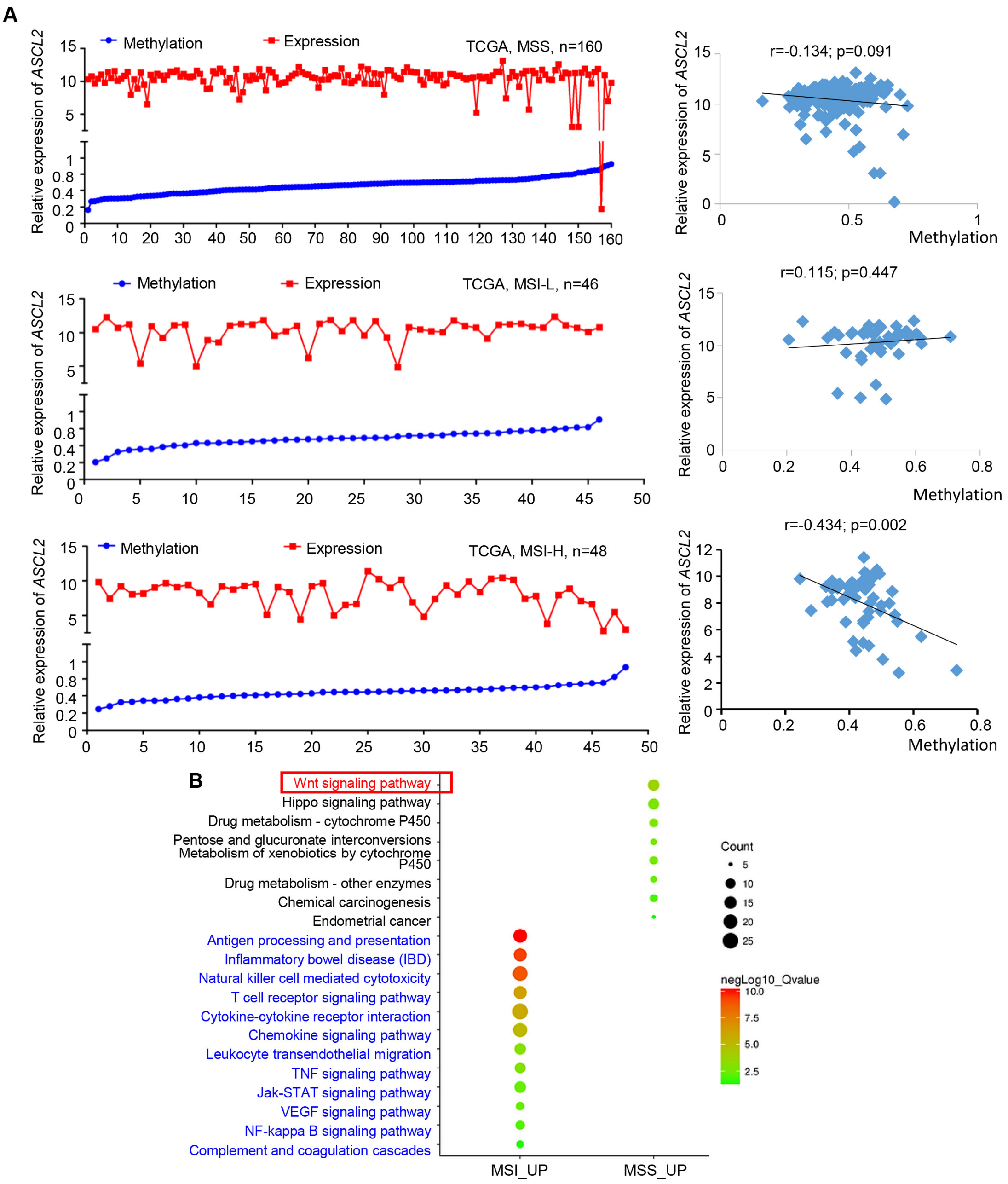

Supplementary Fig. 2 Analysis of *ASCL2* expression patterns in MSS and MSI-H CRCs

(A) Spearman's correlation analysis was used to analyze the correlation between *ASCL2* mRNA expression and methylation in TCGA CRCs database (160 MSS tumors, 46 MSI-Low tumors, and 48 MSI-High tumors). Scatter diagrams represent the correlation coefficient between *ASCL2* and methylation degree. *p<0.05. **p<0.01. (B) Bubble chart shows the correlation analysis of *ASCL2* mRNA expression and signaling pathways in MSS and MSI CRCs.

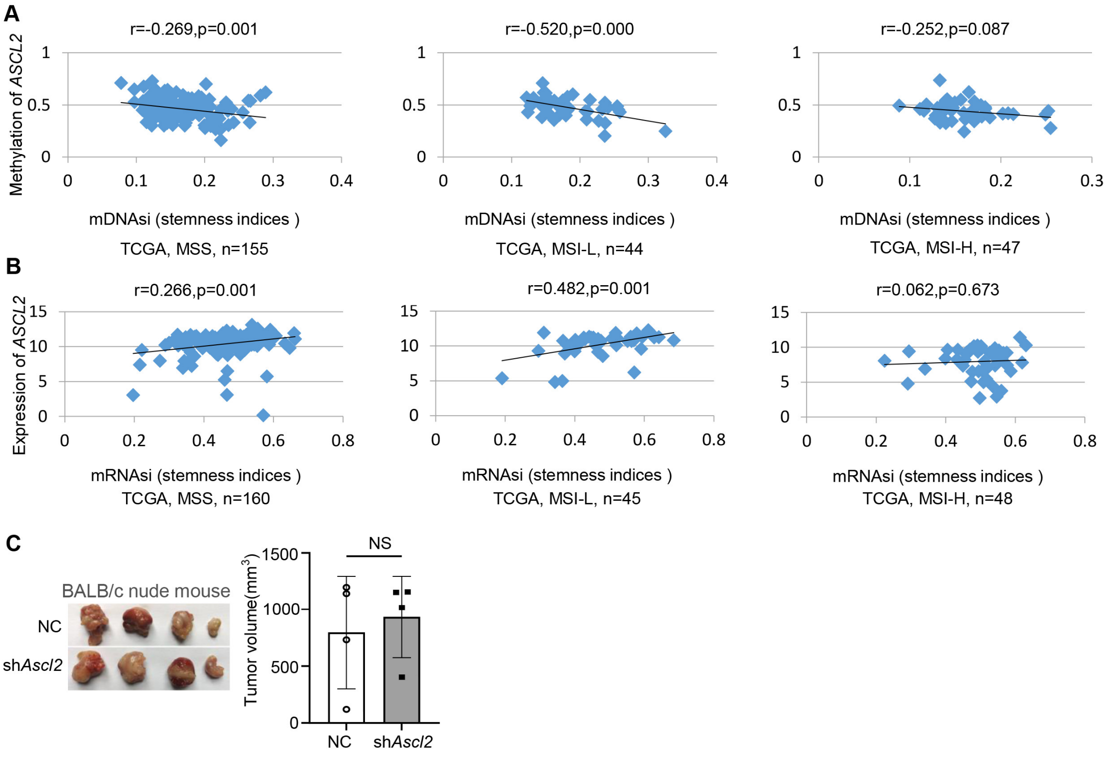

Supplementary Fig. 3 ***ASCL2*** **expression maintains the stemness phenotype without affecting the proliferation of CRC cells**

(A) Spearman's correlation analysis between *ASCL2* methylation degree and stemness indices in TCGA CRCs database (155 MSS tumors, 44 MSI-Low tumors, and 47 MSI-High tumors). (B) Spearman's correlation analysis between *ASCL2* mRNA expression and stemness indices in TCGA CRCs database (160 MSS tumors, 45 MSI-Low tumors, and 48 MSI-High tumors). **p<0.01. (C) Representative images of primary tumor in intestines orthotropic transplantation assay in nude mice. Histograms represent the statistics of tumor volume. NS, no significance.

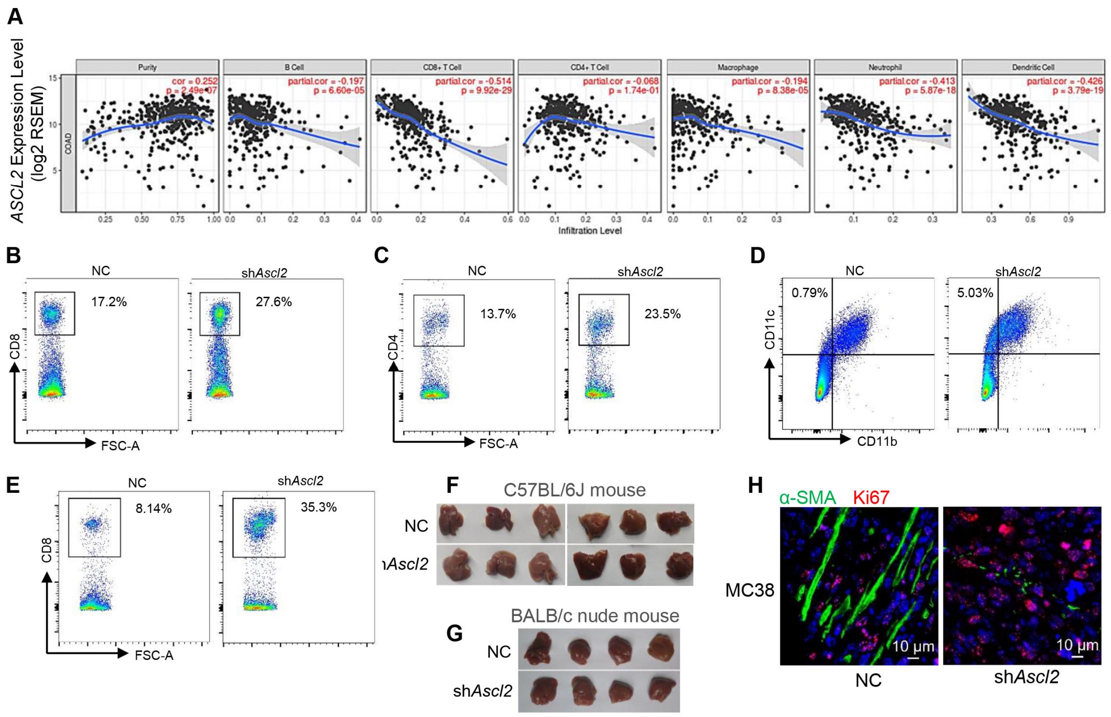

Supplementary Fig. 4

(A) Correlation analysis of ASCL2 mRNA expression with infiltrating immune cells in CRC microenvironment in TIMER 2.0 analysis database. (B-D) Representative images of tumor-infiltrated $CD8^+$ T cells (B), $CD4^+$ T cells (C), and $CD11C^+CD11B^-$ DCs (D) cells profiling using flow cytometry in a subcutaneous xenograft tumor model formed by MC38/NC and MC38/sh*Ascl2* cells. (E) Representative images of tumor-infiltrated $CD8^+$ T cells using flow cytometry in the orthotopic implantation mouse model formed by MC38/NC and MC38/sh*Ascl2* cells. (F-G) Representative images of liver in intestines orthotropic transplantation assay in C57BL/6J mice (F) and nude mice (G). (H) Representative images of mIF for Ki67 (red) and α-SMA (green) in orthotopic implantation tumor tissue. Scale bar, 10μm.

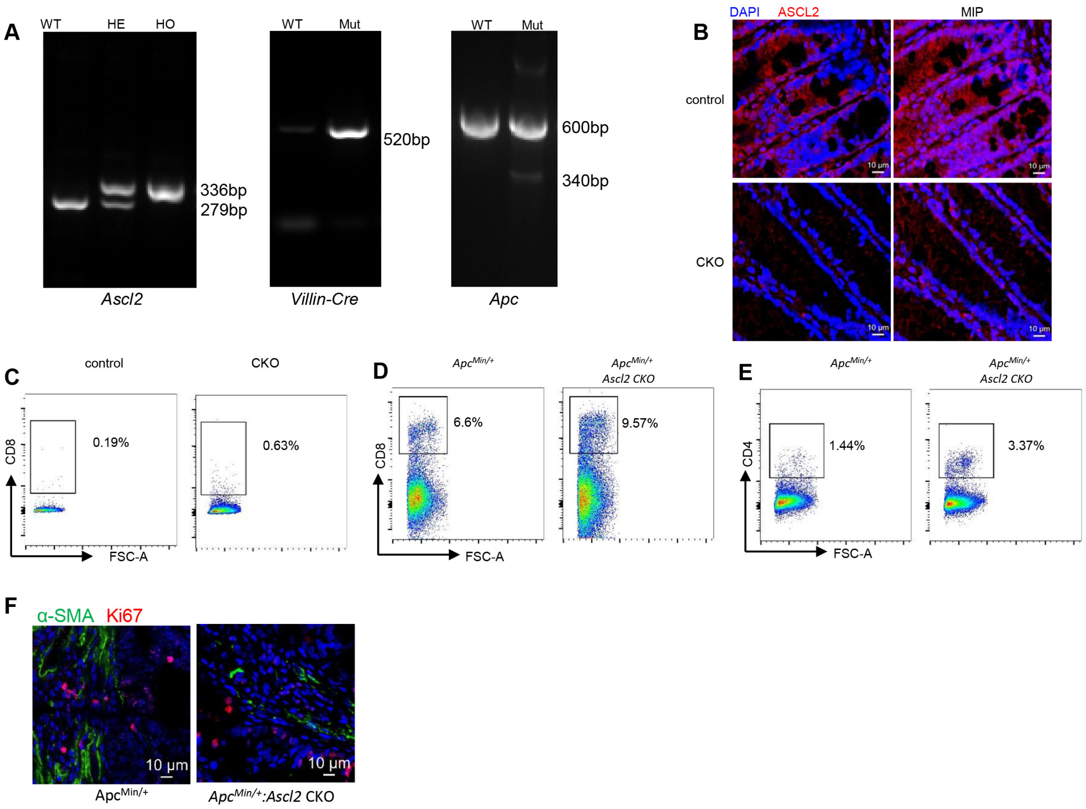

Supplementary Fig. 5 Deletion of intestinal epithelial *Ascl2* promotes inflamed immune microenvironment formation in conditional knockout mice

(A) Gel electrophoresis results of PCR identification. Left: one band with 279bp represents wild type (WT); two bands with 279bp and 336bp represent heterozygous (HE); one band with 336bp represents homozygous (HO). Middle: one band with 520bp represents Cre activity. Right: one band with 600bp represents WT; one band with 340bp represents mutant. (B) Representative images of ASCL2 expression in mice intestinal epithelial tissues. Left: Two-dimensional (2-D) images Right: Three-dimensional (3-D) images are processed by the maximum intensity projection (MIP) method. Scale bars, 10um. (C) Representative images of infiltrated CD8^{+} T cells using flow cytometry in the *Ascl2* CKO mice and control (*Ascl2$^{flox/flox}$*) mice. (D-E) Representative images of infiltrating CD8^{+} T cells (C) and CD4^{+} T (D) cells in the tumor tissue as assayed by flow cytometry in the *Apc$^{Min/+}$* mice and *Apc$^{Min/+}$*;*Ascl2* CKO mice. (F) Representative images of

mIF for Ki67 (red) and α-SMA (green) in intestinal epithelial tissue of *Apc*$^{Min/+}$ mice and *Apc*$^{Min/+}$; *Ascl2* CKO mice.

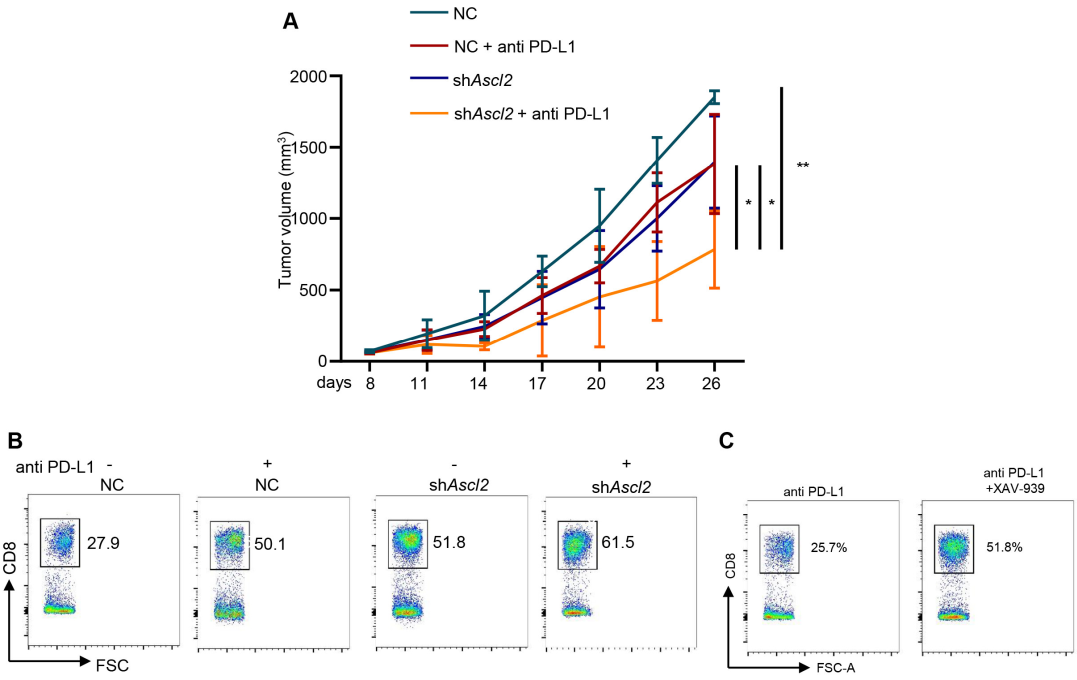

Supplementary Fig. 6 *Ascl2* promotes CRCs progression by inhibiting CD8^{+} T cell infiltration in the tumor microenvironment

(A) Tumor volume curves for each group (n=5). Due to the inevitable factors, the initial tumor volume of the groups was different, and samples that did not fall within the InterQuartile Range (IQR, the distance between 1st quartile and 3rd quartile) are removed. Finally, the tumor volume growth curve of each group (n=5) was obtained. The variances between the groups were similar using the two-way ANOVA for statistics. Error bars, mean ± SD. *p<0.05, **p<0.01.

(B) Representative images of infiltrated CD8^{+} T cells using flow cytometry in the mouse CRC model treated with anti-PD-L1.

(C) Representative images of infiltrated CD8^{+} T cells using flow cytometry in the mouse CRC model treated with XAV939.

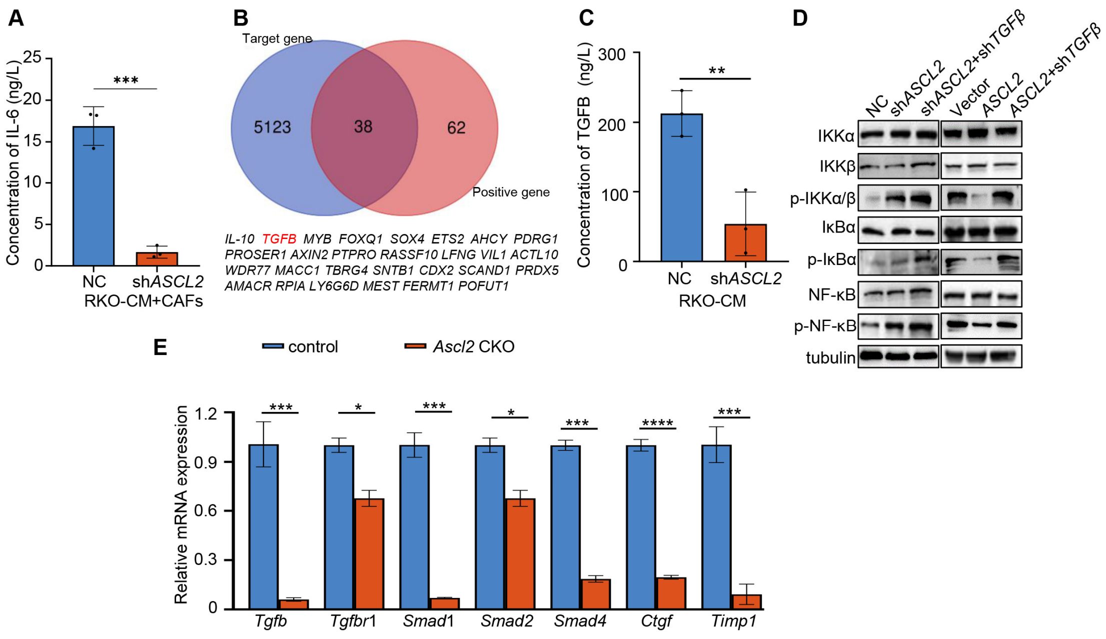

Supplementary Fig. 7 *ASCL2* induces CAFs activation to exclude CD8$^+$ T cells by transcriptionally activating *TGFB*

(A) RKO cells were transfected with sh*ASCL2*, and the supernatant was filtered and collected after 48 hours, followed by coculture with human primary CAFs for 48 hours. IL-6 production was measured by ELISA. Error bars, mean ± SD; ***p<0.001. (B) Venn diagram shows the possible transcription target genes of *ASCL2* by the GTRD database. (C) RKO cells were transfected with sh*ASCL2* for 48 hours before *TGFB* was assayed using ELISA. Error bars, mean ± SD; **p<0.01. (D) Protein expression of NF-κB signaling pathway in the indicated cells. α-Tubulin was used as a loading control. (E) Relative mRNA expression of TGFβ-Smad signaling genes in intestinal epithelial cells in indicated mice. Error bars, mean ± SD; *p<0.05, ***p<0.001, ****p<0.0001.

Supplementary materials and methods

Establishment of stable cell lines and construction of short hairpin RNA plasmid

ASCL2 (homo) lentivirus was purchased from GenePharma (SuZhou, China), the plasmid is LV5(EF-1aF/GFP&Puro). sh*Ascl2* (mouse) lentivirus was purchased from GenePharma, the plasmid is LV3(H1/GFP&Puro).

To knock down ASCL2 or TGFB expression in CRC cells, 2 short hairpin RNA (shRNA) oligonucleotides were respectively cloned into the pLKO.1-puro-GFP to generate *ASCL*2-shRNA or *TGFB*-shRNA. The primers used are below. *ASCL2* shRNA Top Strand: CCGGAGCGCGAGCTACTCGACTTCTCTCGAGAGAAGTCGAGTAGCTCGCGCTTTTTTG; Bottom Strand: AATTCAAAAAAAGCGCGAGCTACTCGACTTCTCTCGAGAGAAGTCGAGTAGCTCGCGC. *TGFB* shRNA Top Strand: CCGGCAAGCAGAGTACACACAGCATCTCGAGATGCTGTGTGTACTCTGCTTGTTTTT; Bottom Strand: AATTAAAAACAAGCAGAGTACACACAGCATCTCGAGATGCTGTGTGTACTCTGCTTG.

Isolation and purification of primary human CRC CAFs

Tumor tissues from CRC patients were washed three times with PBS containing 10U/L penicillin and 100mg/L streptomycin and finely cut into 0.5-1.0 mm fragments. The tissue pieces were evenly spread on the bottom of the culture flask, followed by adding a 2mL DMEM medium containing 10% FBS, 10U/L penicillin, and 100mg/L streptomycin. And then, the flask was placed upside down in the incubator. After 2 hours, the flask was turned over and incubated. The medium was changed for the first time the next day and then every 3 days until the cells covered the

bottom of the bottle (about 2 weeks). CAFs were subcultured and purified by differential attachment methods. Then cells from the third to the eighth generation were used for subsequent experiments

Limiting dilution xenotransplantation assay

The indicated numbers of cells were injected subcutaneously into the flanks of nude mice (n = 6), and the number of tumors formed out of the number of sites injected was scored to determine the frequency of sphere-initiating cells or cancer-initiating cells calculated using the ELDA software (http://bioinf.wehi.edu.au/software/elda/index.html).

Tumorsphere formation assay

The isolated cells at 500 cells/well were cultured in ultra-low attached 6-well plates (Corning, 3471) in a defined stem cell medium. The stem cell medium was Dulbecco's Modified Eagle's medium/F12 medium (Gibco, 11330032) containing 2% B27 supplement (Gibco, 17504044-1), 20 ng/ml of epidermal growth factor (PeproTech, AF-100-15), 20 ng/ml of HGF (PeproTech, 100-18B) and 5 µg/ml of insulin (Sigma, 12584-58-6) for 2 weeks. The diameters of at least 10 tumorospheres were examined under a microscope to calculate the average diameters of tumorospheres. The size of spheres was measured using Fiji.

Orthotopic implantation mouse model

C57BL/6 mice or Balb/C athymic nude mice (female, 6 weeks of age) were injected subcutaneously with 2×10^5 MC38 cells, and animals were sacrificed two weeks later. The

subcutaneous tumors were excised and sliced into small pieces (approximately 1mm^3). The C57BL/6 mice and nude mice of the experimental group were anesthetized with 1% pentobarbital and implanted the tissue fragments on the mucosa of the cecum. The general growth conditions in the experimental mice were observed after surgery to observe whether there were signs of exhaustion. 40 days (C57BL/6 mice) or 70 days (nude mice) after the operation, all mice were sacrificed, and the organs of the intestines, livers, and lungs were removed for observing and photographing. After formalin fixation, paraffin sections were prepared for further experiments.

Histology, immunohistochemistry, immunofluorescence, and confocal microscopy

Colon tissue specimens were fixed with formalin, embedded with paraffin, dehydrated in ethanol, and stained with H&E or Sirius red staining using a picrosirius red staining solution (Phygene, PH1098).

For immunohistochemistry, after treatment with 3% hydrogen peroxide (ZSGB-Bio) to quench the endogenous peroxidase activity, the sections were submerged into citrate buffer and high-pressure boiled for antigenic retrieval, followed by incubation with goat serum (ZSGB-Bio) to block the nonspecific binding for 30 min. Primary antibodies were incubated with the sections overnight at 4°C. After washing, the tissue sections were treated with biotinylated anti-rabbit secondary antibody (ZSGB-Bio), followed by further incubation with streptavidin-horseradish peroxidase complex (ZSGB-Bio). The tissue sections were incubated with 3,3-diaminobenzidine (ZSGB-Bio-Bio) and counterstained with hematoxylin (ZSGB-Bio), dehydrated, and mounted.

For immunofluorescence, tumor tissue sections were boiled in 10 mM sodium citrate (pH 6.0), for antigen retrieval after deparaffinization. After normal goat serum (ZSGB-Bio) blocking for 30

minutes at 37°C, the sections were incubated with primary antibody for 16 h at 4°C. The corresponding fluorochrome-labeled secondary antibody was applied for 30 minutes at room temperature in the dark, and the samples were washed and then mounted with DAPI to label nuclear (Sigma, D9542). Following washes in PBS, coverslips were mounted onto slides with Vectashield (Solarbio) and sealed using transparent nail polish. Images were acquired on a confocal laser-scanning microscope (Zeiss LSM880, Olympus FV3000).

ELISA

The cell culture medium was changed after CRC cells were transfected with sh*ASCL2* plasmid for 48 h. The supernatants were collected after three days and filtered with an ultrafiltration tube (Millipore, UFC8030). The concentrations of cytokines in culture media were evaluated using human TGFβ uncoated ELISAs (ELGbio, EW0113Hu) according to the manufacturer's instructions.

The above-filtered concentrations were added to the CAFs medium three times over 5 days. After the last addition of the concentrate and incubation for two days, the medium of the CAFs was replaced by the usual culture. After culture for two days, the supernatant of CAFs was collected and filtered through an ultrafiltration tube (Millipore, UFC8003). The filtered concentrate was detected with an IL-6 ELISA kit (MEIMIAN, 0049H2).

Chromatin Immunoprecipitation

ChIP assays were carried out using a kit (ACTIVE MOTIF, ChIP-IT Express, 53008). Briefly, cells (2×10^7) in a 10-cm culture dish were treated with 1% formaldehyde to crosslink chromatin-

associated proteins to DNA. The cell lysates were subjected to ultrasound for 9–10 sets of 10-s pulses at 40% output to shear the DNA into fragments between 200 and 1000 bps. Equal cell lysates were incubated with 1 μg of anti-ASCL2 antibody (Millipore, MAB4418) and anti-IgG antibody as a negative control. All the above chromatin supernatants were incubated with 20 μL of magnetic protein G beads overnight at 4 °C with rotation. On the second day, the protein-DNA complexes were reversed and purified to obtain pure DNA. The human *TGFB* promoter was amplified with RT-PCR.

RNA isolation and real-time quantitative-PCR

The total RNA was performed using SevenFast total RNA extraction Kit (SM132, SEVEN Biotech) in accordance with the manufacturer's protocols and reverse-transcribed into cDNA using One-Step gDNA Removal (AU341-02, Transgen). Real-time PCR was performed using SYBR qPCR Master Mix (Q711, Vazyme) with an Applied Biosystem 7500 instrument (Applied Biosystems). Relative expression to *GAPDH* was determined using the $2^{-\Delta\Delta Ct}$ method. The primers used are shown in Supplementary Table 2.

Western blot

Total protein was extracted using radioimmunoprecipitation assay buffer (50 mmol/L Tris-HCl pH 8.0, 150 mmol/L NaCl, 0.5% Na-deoxycholate, 0.1% sodium dodecyl sulfate, 1mM PMSF). Protein concentration was determined by BCA Protein Assay Kit (KGP902, KeyGEN). Equal amounts of protein lysates were separated by SDS-PAGE gel and electrotransferred to PVDF membrane. After blocking in 5% milk-TBST for 1 hour at room temperature (RT), the membrane

was incubated with primary antibodies (Supplementary Table 1) at 4 °C overnight and secondary antibodies at RT for 45min. Images were acquired using a ChemiDoc™ Imaging System (Bio-Rad, USA).

Side population (SP) cell staining and analyses

CRC cells were washed with PBS, and digested by trypsin, and 1×10^6/ml cells were collected. Subsequently, 5 μg/ml Hoechst 33342 (Thermo Fisher, 62249) was added at 37°C for 90 min. In total, 100 μg/ml Verapamil (Sigma-Aldrich; V4629) was added in advance as the negative control. Upon staining, the cells were added to 1 μg/ml propidium iodide and sorted by flow cytometry. Cells were then washed and analyzed by flow cytometry on a MoFlo XDP instrument with a UV laser and Hoechst blue (450/50nm) and Hoechst red (670/30nm) bandpass filters. The SP and non-SP cells were collected and calculated the percentages.

Chromatin immunoprecipitation (ChIP)

ChIP assays were carried out using EZ-ChIP (Millipore, 17-371) kit according to the manufacturer's instructions. Briefly, approximately 5×10^6 cells were treated with 1% formaldehyde for 10 min to crosslink chromatin-associated proteins to DNA and then added glycine to quench unreached formaldehyde. Cells were collected and sonicated to shear the DNA to 200–1000-bp fragments. Then the lysates were cleared by centrifugation at 12000 rpm for 10 min at 4 °C. 60μL of protein G agarose was added to the lysates and incubated for 1 hour at 4 °C to preclear the chromatin. The precleared lysates were incubated with anti-ASCL2 antibody (Millipore, MAB4418) or normal mouse immunoglobulin G as a negative control overnight at

4 °C with rotation. Immunoprecipitation of the DNA–protein complexes was proceeded with 60μL of protein G agarose for 1 hour at 4 °C and followed by isolation of the DNA. After reverse cross-link of protein/DNA complexes to free DNA, RT-PCR was performed. The primers used are below. Forward primer: 5‘-TTTGCCATGTGCCCAGTA-3’; Reverse primer:

5’-CCACCACCCACGAAAGC-3’.

Luciferase reporter assay

5×10^4 cells were seeded in 24-well plates and settled for 48 hours. 1.5μg of *ASCL2* luciferase reporter plasmid and the control-luciferase plasmid, plus 0.15μg of PRL-SV40 plasmid, were respectively transfected into cells according to the manufacturer's protocols. Luciferase activity was measured using Dual Luciferase Reporter Assay Kit (Promega, E1960) and normalized to Renilla luciferase gene expression. All the experiments were performed in triplicate.

Computational analysis of immunofluorescent and immunohistochemistry images

All images were analyzed using Fiji. T cell density per area was analyzed in tissues after CD8 or CD4 staining. Areas were drawn using Fiji. After drawing the regions of interest (ROIs), CD8 or CD4 signals were revealed, and T cells were counted manually. The area was calculated by FiJi after scaling images. The density of T cells was calculated as the number of T cells/area (mm^2). Three or more random microscopic fields were analyzed for each sample.

Bioinformatics analysis

Data on *ASCL2* expression, stemness index analysis, methylation analysis, and overall survival

analysis were obtained from the Gene Expression Omnibus (GEO) database (GSE13294, GSE71187, and GSE41258) and TCGA database. Prediction of *ASCL2* transcriptional target genes was obtained from the GTRD (Gene Transcription Regulation Database).
